# BRD3OS Dysregulation in Antiphospholipid Syndrome: Integrative Network and RNA Structural Analysis of m6A-Related Candidate Regions

**DOI:** 10.3390/biom16091263

**Published:** 2026-09-01

**Authors:** Carlos A. Guzmán-Martín, Yaneli Juárez-Vicuña, Rafael Bojalil, Evelyn Aranda-Cano, Mario Peña-Peña, Yamnia Q. Alvarez-Alvarez, Fengyang Huang, Javier González-Ramírez, Laura Aline Martínez-Martínez, Fausto Sánchez-Muñoz

**Affiliations:** 1 Doctorado en Ciencias Biológicas y de la Salud, Universidad Autónoma Metropolitana, Mexico City 04960, Mexico; gmcarlos93@gmail.com; 2 Departamento de Inmunología, Instituto Nacional de Cardiología Ignacio Chávez, Mexico City 14080, Mexico; yaneli2608@hotmail.com; 3 Departamento de Atención a la Salud, Universidad Autónoma Metropolitana, Mexico City 04960, Mexico; rafaelbojalil@gmail.com; 4 Departamento de Reumatología, Hospital Central Militar, Secretaría de la Defensa Nacional, Mexico City 11200, Mexico; canoevelyn12@gmail.com; 5 Departamento de Fisiología, Instituto Nacional de Cardiología Ignacio Chávez, Mexico City 14080, Mexico; marionutricion2017@gmail.com (M.P.-P.); yamniaalvarezalvarez@gmail.com (Y.Q.A.-A.); 6 Laboratorio de Investigación en Obesidad y Asma, Hospital Infantil de México Federico Gómez, Mexico City 06720, Mexico; huangfengyang@gmail.com; 7 Laboratorio de Biología Molecular, Facultad de Enfermería, Universidad Autónoma de Baja California, Campus Mexicali, Mexicali 21100, Mexico; javier.gonzalez.ramirez@uabc.edu.mx; 8 Departamento de Reumatología, Instituto Nacional de Cardiología Ignacio Chávez, Mexico City 14080, Mexico

**Keywords:** antiphospholipid syndrome, BRD3OS, LINC00094, long non-coding RNA, m6A, epitranscriptomics, RNA structure, DRACH motifs, RNA accessibility, peripheral blood mononuclear cells

## Abstract

Antiphospholipid syndrome (APS) is an autoimmune disorder characterized by thrombotic and inflammatory manifestations whose molecular regulatory mechanisms remain incompletely understood. Long non-coding RNAs (lncRNAs) and N6-methyladenosine (m6A)-related regulation are increasingly recognized as components of immune gene regulation, but their involvement in APS remains poorly characterized. This study investigated BRD3OS (LINC00094) expression in APS and explored its molecular and predicted structural context in relation to m6A-associated regulation. An exploratory case–control study was conducted using an initial lncRNA PCR-array discovery cohort followed by targeted RT-qPCR validation in an independent cohort. Candidate prioritization incorporated multiple expression and technical features and was evaluated through sensitivity analyses. BRD3OS expression, selected m6A regulators (METTL3, METTL14, WTAP, and FTO), inflammatory mediators, and global m6A abundance in total peripheral blood mononuclear cell (PBMC) RNA were evaluated. Bioinformatic network analysis was used to contextualize BRD3OS within APS- and m6A-related molecular systems. RNAfold and RNAplfold were used to characterize the predicted structural context and accessibility of DRACH consensus motifs, with additional analyses evaluating fragment-boundary and composite-score robustness. BRD3OS was significantly downregulated in PBMCs from patients with APS in the independent validation cohort. METTL3, METTL14, and WTAP expression was also reduced, whereas global m6A levels in total PBMC RNA were increased. These observations indicate concurrent alterations in BRD3OS expression and the broader m6A-related molecular environment but do not establish transcript-specific methylation of BRD3OS. Bioinformatic network analysis placed BRD3OS within predicted RNA-centered regulatory relationships relevant to APS. DRACH motifs exhibited heterogeneous predicted structural accessibility, with unpaired structural environments showing greater RNAplfold-derived accessibility than paired regions. Quantitative accessibility estimates were highly concordant across overlapping transcript fragments, although sensitivity analyses indicated that the identity of individual highest-ranked candidates depended on the weighting scheme. BRD3OS downregulation represents a reproducible molecular finding in APS. Concurrent alterations in global m6A abundance and selected m6A regulators suggest broader epitranscriptomic dysregulation; however, these measurements cannot establish m6A modification of BRD3OS or a causal relationship between these observations. Structural and network analyses therefore provide a hypothesis-generating framework for prioritizing candidate regions and interactions for future transcript-specific methylation mapping and functional validation.

## 1. Introduction

Antiphospholipid syndrome (APS) is a systemic autoimmune disorder characterized by recurrent thrombosis and obstetric morbidity in the presence of antiphospholipid antibodies [1,2]. Beyond its clinical manifestations, APS is increasingly understood as a complex immunothrombotic condition involving chronic low-grade inflammation, endothelial dysfunction, and aberrant activation of innate immune pathways [3,4]. Central to its pathophysiology is the interplay between proinflammatory signaling cascades such as those mediated by Toll-like receptors and NF-κB and a persistent procoagulant state, yet the upstream regulatory mechanisms orchestrating these processes remain incompletely defined [5]. In recent years, long non-coding RNAs (lncRNAs) have emerged as critical regulators of immune homeostasis and gene expression [6]. These transcripts, exceeding 200 nucleotides in length and lacking protein-coding potential, modulate cellular processes through diverse mechanisms, including chromatin remodeling, transcriptional control, and post-transcriptional regulation [7]. Several lncRNAs have been implicated in autoimmune diseases, where they contribute to immune cell activation, cytokine production, and disease progression [8,9]. However, the functional landscape of lncRNAs in APS remains largely unexplored, representing a significant gap in our understanding of disease-specific regulatory networks.

Among these, the lncRNA BRD3OS (also known as LINC00094) has been proposed as a potential modulator of inflammatory signaling, although its biological role remains poorly characterized [10,11]. Emerging evidence suggests that BRD3OS may participate in transcriptional regulatory circuits linked to immune activation and cellular stress responses, positioning it as a candidate molecule in immunoinflammatory diseases [12]. Nevertheless, its expression patterns, regulatory context, and functional implications in APS have not been systematically investigated.

Epitranscriptomic modifications, particularly N6-methyladenosine (m6A), add an additional layer of complexity to RNA regulation. m6A is the most abundant internal modification in eukaryotic mRNA and is also present in non-coding RNAs. Its deposition, recognition, and removal involve coordinated regulatory proteins, including methyltransferase components (“writers”) such as METTL3, METTL14, and WTAP; demethylases (“erasers”) such as fat mass and obesity-associated protein (FTO) and AlkB homolog 5, RNA demethylase (ALKBH5); and m6A-binding proteins (“readers”) [13,14]. Through these regulatory systems, m6A can influence RNA stability, processing, localization, and translation. Altered m6A-related regulation has also been implicated in immune and inflammatory responses [15].

Long non-coding RNAs can themselves contain m6A modifications, which may influence their structure, stability, localization, or molecular interactions [16]. However, the presence of a canonical DRACH consensus motif alone does not establish that a nucleotide is methylated. m6A deposition is context dependent, and local RNA structural accessibility may contribute to the recognition or exposure of candidate motifs [17]. Consequently, computational identification of DRACH motifs and prediction of their structural accessibility can be used to prioritize candidate regions for subsequent experimental interrogation but cannot substitute for transcript-specific m6A mapping.

This distinction is particularly relevant in APS, where neither the m6A status of BRD3OS nor the relationship between global RNA methylation and BRD3OS expression has been established. Moreover, measurements of global m6A abundance in total RNA reflect the aggregate RNA pool and therefore cannot be attributed to an individual transcript. Accordingly, alterations in BRD3OS expression, global m6A abundance, and expression of selected m6A regulators should be considered complementary but analytically distinct observations unless a direct molecular relationship is experimentally demonstrated.

In this study, we first evaluated BRD3OS expression in peripheral blood mononuclear cells (PBMCs) from patients with APS using an exploratory discovery cohort followed by targeted validation in an independent cohort. We then characterized the broader m6A-related molecular context by measuring global m6A abundance in total PBMC RNA and the expression of selected m6A regulatory genes and inflammatory mediators. Finally, we integrated public interaction resources with RNA secondary-structure and local-accessibility predictions to characterize the in silico regulatory and structural context of BRD3OS. Specifically, we examined the distribution and predicted accessibility of DRACH motifs as candidate regions potentially compatible with m6A-associated regulation. These computational analyses were designed as hypothesis-generating approaches and do not demonstrate methylation of BRD3OS, identify experimentally validated m6A sites, or establish a causal relationship between BRD3OS expression and the global m6A alterations observed in APS.

## 2. Materials and Methods

### 2.1. Study Design and Participants

This study was designed as an exploratory case–control investigation embedded within a multi-layered analytical framework integrating experimental and computational approaches to characterize BRD3OS (LINC00094) dysregulation and its broader m6A-related molecular and predicted structural context in antiphospholipid syndrome (APS). In the discovery phase, peripheral blood samples were obtained from 7 patients with a confirmed APS diagnosis, established according to internationally accepted clinical and laboratory criteria, and 7 age-matched healthy controls; all participants provided written informed consent, and the protocol was approved by the institutional ethics committee (registry number: 23-1399; approval date: 28 September 2023) in accordance with the Declaration of Helsinki. An independent validation cohort comprising 35 APS patients and 24 healthy controls was subsequently included for targeted expression analysis.

### 2.2. PBMC Isolation and Total RNA Extraction

Peripheral blood (10 mL) was collected into EDTA tubes and processed within 2 h of venipuncture. Peripheral blood mononuclear cells (PBMCs) were isolated by density-gradient centrifugation using Histopaque®-1077 (Sigma-Aldrich, St. Louis, MO, USA) washed twice with phosphate-buffered saline (PBS), and counted using a hemocytometer. Cell pellets were stored at −80 °C until RNA extraction.

Total RNA was extracted from PBMC pellets using a Direct-zol RNA Microprep Kit (Zymo Research, Irvine, CA, USA; Cat. No. R2062), following the manufacturer’s protocol. RNA yield and purity were assessed with a NanoDrop 2000c spectrophotometer (Thermo Scientific, Wilmington, DE, USA), and only samples with an A260/280 ratio between 1.8 and 2.1 were included. RNA concentration was adjusted to ensure sufficient input for downstream applications.

PBMCs were analyzed as an unsorted bulk cell population; no cell-subset purification or cell-composition profiling was performed. Consequently, the expression measurements reported in this study represent aggregate PBMC-level signals and cannot be assigned to specific immune-cell populations.

### 2.3. cDNA Synthesis

Complementary DNA (cDNA) was synthesized from 500 ng of total RNA using the QuantiTect Reverse Transcription Kit (QIAGEN, Hilden, Germany; Cat. No. 205311), which includes genomic DNA elimination and reverse-transcription steps. Reverse transcription was performed according to the manufacturer’s instructions in a total reaction volume of 20 µL. The resulting cDNA was diluted 1:5 with nuclease-free water and stored at −20 °C until quantitative PCR analysis.

### 2.4. lncRNA Expression Profiling Using RT^2^ PCR Array

Peripheral blood mononuclear cells were obtained from 7 patients diagnosed with APS and 7 healthy controls. Total RNA was extracted and analyzed using a commercially available RT^2^ lncRNA PCR Array (QIAGEN) targeting 84 lncRNAs associated with inflammation and autoimmunity. Raw cycle threshold (Ct/Cq) values were exported for downstream analysis.

### 2.5. Data Preprocessing and Normalization

Raw quantification cycle (Cq) values obtained from the lncRNA PCR array were processed using R (version 4.5.2) (R Foundation for Statistical Computing, Vienna, Austria). Initial preprocessing included removal of non-informative entries such as annotation rows and control features, standardization of sample identifiers, and conversion of all measurements to numeric format. Non-detected values (reported as “N/A” or equivalent) were treated as missing values and excluded from downstream calculations without imputation.

To ensure technical robustness, only lncRNAs detected in at least four samples in either study group were retained for further analysis. This filtering step was applied to minimize the influence of sparsely detected transcripts and reduce noise associated with low-abundance signals. Normalization was performed using the set of housekeeping genes included in the array (ACTB, B2M, RPLP0, RN7SK, and SNORA73A). For each sample, the mean Cq value of the housekeeping genes was calculated and used as a reference. Relative expression levels were computed using the delta Ct method, defined as the difference between the Cq value of each target transcript and the corresponding sample-specific mean housekeeping Cq. Group-level expression differences were summarized using delta delta Ct values, calculated as the difference between the mean delta Ct in the APS group and the mean delta Ct in the control group. Fold changes were derived from delta delta Ct values using the standard transformation. Quality control of housekeeping gene expression was performed by evaluating their distribution across samples and groups, ensuring stability and absence of systematic bias prior to normalization.

### 2.6. Exploratory Differential Expression Analysis

Differential expression in the discovery cohort was evaluated primarily using the limma package in R. Limma was applied to normalized ΔCt values using a group-based design matrix (APS vs. control), with empirical Bayes moderation implemented through eBayes(). For interpretation, ΔCt-derived coefficients were converted to expression-direction estimates so that positive and negative fold-change values consistently reflected relative expression between groups. Multiple-testing correction was performed using the Benjamini–Hochberg procedure.

Because the discovery cohort was intentionally small (*n* = 7 per group), the analysis was considered exploratory. No transcript met the conventional false-discovery-rate threshold after Benjamini–Hochberg correction. Unadjusted two-sample comparisons of ΔCt values were additionally calculated as descriptive measures to characterize individual transcript behavior; these tests were not used as an independent criterion for declaring differential expression and did not supersede the limma analysis or adjusted *p*-values.

Accordingly, discovery-phase results were not interpreted as confirmatory evidence of differential expression. Instead, they were used to generate candidates for subsequent prioritization and independent experimental validation.

### 2.7. Multi-Parameter Candidate Prioritization

Because no lncRNA reached statistical significance after multiple-testing correction in the discovery cohort, candidate selection was treated as a prioritization procedure rather than as confirmatory statistical inference. Transcripts were ranked by integrating complementary technical and expression-related features intended to identify candidates with reproducible amplification characteristics and potentially relevant expression patterns for subsequent independent validation.

For each of the 69 evaluable lncRNAs, five components were calculated. Detectability was defined as the proportion of non-missing Ct observations across discovery samples. Expression variability was represented by the standard deviation of ΔCt values across samples and transformed inversely so that lower variability received a higher score. Effect size was defined as the absolute difference in mean ΔCt between APS and control groups. Mean amplification level was represented by the mean Ct across available discovery samples and was transformed inversely so that lower Ct values received higher scores. Exploratory statistical support was represented by −log10 of the nominal *p*-value obtained from the primary limma analysis. Each component was min–max normalized to a 0–1 scale, with higher values consistently representing more favorable characteristics for candidate prioritization.

A continuous composite score was then calculated using the following investigator-defined weighting scheme: detectability, 0.30; inverse variability, 0.20; effect size, 0.20; inverse mean Ct, 0.20; and exploratory statistical support, 0.10. These heuristic weights were selected to place the greatest emphasis on reliable detection while balancing technical stability, magnitude of the observed between-group difference, amplification characteristics, and nominal statistical support. The composite score was not intended to represent a fitted predictive model, a probability of differential expression, or a substitute for multiple-testing-adjusted statistical inference.

To determine whether candidate prioritization was excessively dependent on the investigator-defined weighting scheme, three complementary sensitivity analyses were performed. First, rankings were recalculated using equal weighting of all five components. Second, leave-one-component-out analyses were performed by sequentially excluding each component and recalculating the score using the remaining original weights rescaled to sum to 1. Third, 100,000 alternative weight vectors were sampled from a Dirichlet distribution centered on the original weight vector (0.30, 0.20, 0.20, 0.20, and 0.10), using a concentration parameter of 50. Each sampled weight vector was constrained to sum to 1.

Ranking robustness was evaluated by examining the position of BRD3OS across alternative scoring schemes. For the Monte Carlo analysis, its median rank, interquartile range, and proportion of simulations in which it remained among the top 10 candidates were calculated. These analyses were used exclusively to evaluate stability of candidate prioritization and were not interpreted as additional evidence of differential expression.

BRD3OS (LINC00094) was advanced to independent RT-qPCR validation because it combined reliable detectability, favorable amplification characteristics, an expression pattern of biological interest, and stable prioritization across alternative weighting configurations. Selection through this framework was considered hypothesis-generating; evidence supporting BRD3OS dysregulation in APS was therefore based primarily on its subsequent evaluation in the independent validation cohort rather than on the discovery ranking itself.

Complete candidate-prioritization results and sensitivity analyses are provided in Supplementary Data S1 and summarized in Supplementary Table S1.

### 2.8. Candidate Selection and Validation

Candidate lncRNAs were prioritized based on a composite ranking framework integrating detectability, expression consistency, and technical robustness rather than statistical significance alone. LINC00094 (BRD3OS) was selected as the primary candidate due to its complete detectability across samples, stable amplification profile, and suitability for downstream validation, despite the absence of statistically significant differential expression in the discovery cohort.

Validation was conducted in an independent cohort using targeted reverse transcription quantitative PCR (RT–qPCR). In the validation cohort, additional targets including epitranscriptomic regulators (METTL3, METTL14, WTAP, and FTO) and inflammatory mediators (IL6, TNF, and TLR4) were quantified using the same RT–qPCR framework. Reactions were performed using the QuantiNova SYBR Green PCR Kit (QIAGEN, Venlo, The Netherlands; Cat. No. 208052) in a final volume of 10 µL, containing 1× QuantiNova SYBR Green Master Mix, 0.3 µM of each primer, and 2 µL of diluted cDNA template. Primer pairs were designed using NCBI Primer-BLAST (National Center for Biotechnology Information, Bethesda, MD, USA; Primer3 version 2.5.0) to preferentially span exon–exon junctions, minimizing genomic DNA amplification, and were synthesized by Integrated DNA Technologies (Coralville, IA, USA).

Thermal cycling was carried out with an initial enzyme activation step at 95 °C for 2 min, followed by 40 amplification cycles (95 °C for 5 s and 60 °C for 30 s). A post-amplification melting-curve analysis was systematically performed to verify amplification specificity and exclude non-specific products or primer–dimer formation. All reactions were performed in technical duplicates, and no-template controls were included in each run to monitor potential contamination. Expression data were normalized using GAPDH and RPLP0 as endogenous reference genes. Reference-gene stability in the validation cohort was evaluated directly from their Cq distributions. Neither RPLP0 nor GAPDH showed a significant between-group shift in Cq values, and the mean Cq derived from both reference genes was likewise comparable between APS patients and controls. The two reference genes also showed strong concordance across samples. These quality-control findings supported their combined use for normalization. Relative expression levels were calculated using the 2^−ΔΔCt method, with the mean expression of the control group serving as the calibrator.

### 2.9. Quantification of Global m6A RNA Methylation

Global N6-methyladenosine (m6A) levels were quantified in total RNA extracted from PBMCs using the EpiQuik m6A RNA Methylation Quantification Kit (Colorimetric) (Epigentek Group Inc., Farmingdale, NY, USA; Cat. No. P-9005), strictly following the manufacturer’s instructions. Briefly, 200 ng of RNA per sample were bound to assay wells, incubated with capture and detection antibodies specific for m6A, and developed with a colorimetric substrate. Absorbance was measured at 450 nm using a microplate reader, and absolute m6A content (ng) was calculated from a standard curve supplied with the kit and normalized to RNA input.

### 2.10. Integrative Network Construction and Multi-Layer Data Integration

An integrative bioinformatic framework was implemented to characterize the regulatory landscape surrounding BRD3OS (LINC00094) in APS. The pipeline combined experimental data with curated public resources to generate both an exploratory network and a high-confidence publication-ready subnetwork. APS-associated genes were retrieved from the Comparative Toxicogenomics Database (CTD; Disease ID: MESH:D016736) and DisGeNET. Gene symbols were standardized to HGNC nomenclature, harmonized across datasets, and duplicate entries were removed. A consensus APS gene set was defined based on the number of supporting sources per gene.

RNA-centered interactions involving BRD3OS were obtained from the RNAInter database, including RNA–RNA and RNA–protein interactions. Interactions were processed in R and annotated according to functional categories (RNA-binding proteins, transcription factors, coding RNAs, and long non-coding RNAs). For exploratory analyses, all interactions were retained, whereas for the publication network a stringent filtering strategy was applied, retaining only interactions with confidence scores ≥ 0.25 and excluding low-information interaction types and small RNA species (e.g., tRFs and miRNAs). To incorporate epitranscriptomic regulation, experimentally validated m6A regulatory interactions were obtained from RM2Target v2.0; only interactions involving canonical m6A machinery (writers, readers, and erasers) were retained, and evidence was restricted to validated interactions for the publication-level network. Protein–protein interaction (PPI) data were integrated using STRING v12.0, restricting to human proteins (Homo sapiens). For exploratory networks, interactions with combined scores ≥ 400 were included, whereas for publication-level analyses a more stringent threshold (combined score ≥ 700) was applied. Only interactions among APS genes, m6A regulators, BRD3OS interactors, and experimentally validated genes were retained.

### 2.11. Network Assembly and Topological Analysis

All interaction layers derived from RNAInter, RM2Target, and STRING were merged into a unified directed network using the igraph and tidygraph frameworks in R. Nodes were annotated according to molecular class, including lncRNAs, protein-coding genes, and m6A-related writers, readers, and erasers, as well as APS relevance where applicable.

Network topology was characterized using standard centrality measures, including degree, betweenness, closeness, and eigenvector centrality. To facilitate prioritization within the integrated network, a composite node score was calculated by combining APS-association evidence, molecular annotation, connectivity to BRD3OS, available experimental information from the present cohort, differential-expression direction and statistical support where applicable, and network-centrality measures.

The resulting score was used to prioritize nodes within the integrated BRD3OS–APS–m6A-related network for visualization and exploratory interpretation. Network prioritization was considered hypothesis-generating and was not interpreted as evidence of direct physical interaction, regulatory causality, pathway activation, or experimentally validated functional relationships in APS.

### 2.12. Network Integration and Filtering

A multi-layer network was constructed including BRD3OS, APS-associated genes, RNA interactors, m6A-related genes, and protein interactions. To reduce network complexity and enhance interpretability, the network was filtered to retain nodes associated with APS, nodes interacting with BRD3OS, and nodes related to m6A regulation. Protein–protein interactions were retained as contextual background but visually down-weighted. Network visualization was performed using igraph and ggraph, with layout based on the Fruchterman–Reingold algorithm.

### 2.13. Functional Enrichment Analysis

Functional enrichment analysis was performed using the clusterProfiler package, querying Gene Ontology (GO), KEGG pathways, Reactome, and MSigDB Hallmark gene sets. *p*-values were adjusted using the Benjamini–Hochberg method. Enrichment analyses were interpreted as exploratory and hypothesis-generating.

### 2.14. Structural Analysis of BRD3OS

#### 2.14.1. Transcript Selection and Fragmentation

Structural analysis was performed on the BRD3OS transcript ENST00000603928. The sequence was divided into three overlapping fragments (1–3000 nt, 2501–5500 nt, and 5001–5682 nt) to improve computational tractability while preserving continuity between adjacent regions. The resulting dataset comprised 120 fragment-level DRACH observations corresponding to 106 unique transcript-level positions.

Because fragment-wise folding may introduce boundary-dependent structural effects, adjacent fragments contained 500-nt overlapping regions (nt 2501–3000 and 5001–5500). DRACH positions represented independently in two overlapping fragments were matched using transcript-level coordinates to assess the consistency of their structural predictions. Fourteen DRACH positions were independently represented in adjacent fragments. Cross-fragment consistency of RNAplfold-derived accessibility metrics and composite accessibility scores was evaluated using Spearman rank correlation, with Pearson correlation additionally calculated for the composite score. Agreement of discrete RNAfold centroid-derived annotations was evaluated as the proportion of duplicated positions retaining the same paired/unpaired status and local structural-context classification. These analyses were used to assess potential boundary sensitivity introduced by fragment-wise folding.

#### 2.14.2. DRACH Motif Detection

Motifs consistent with the canonical m6A consensus sequence DRACH were identified using the pattern [AGU][AG]AC[ACU], with the central adenosine defined as the candidate position. Motif coordinates were mapped at both fragment and transcript levels. Identification of a DRACH motif was considered a sequence-based candidate criterion and was not interpreted as evidence of m6A modification.

#### 2.14.3. RNA Secondary Structure Prediction

Secondary structure was predicted using RNAfold from the ViennaRNA package version 2.7.2, and centroid structures were used for downstream structural annotation. Each DRACH observation was classified according to its predicted paired/unpaired status and local structural context, including hairpin loop, internal loop/bulge, or stem.

#### 2.14.4. RNA Accessibility Analysis

Local RNA accessibility was calculated using RNAplfold with the parameters RNAplfold -W 150 -L 100 -u 10. Accessibility probabilities were extracted for single-nucleotide, 5-nt, and 10-nt windows. For each DRACH observation, exact accessibility values at the motif-centered position (exact_u1, exact_u5, and exact_u10) and corresponding mean accessibility values across a ±10-nt local neighborhood (mean_u1_pm10, mean_u5_pm10, and mean_u10_pm10) were retained.

#### 2.14.5. Composite Accessibility Score

The six RNAplfold-derived metrics were integrated into a composite accessibility score intended to summarize predicted local RNA exposure. The raw composite score was calculated as:

Composite score = 0.35(exact_u1) + 0.25(exact_u5) + 0.10(exact_u10) + 0.15(mean_u1_pm10) + 0.10(mean_u5_pm10) + 0.05(mean_u10_pm10).

Greater weights were assigned to accessibility estimates closest to the motif-centered nucleotide because these measurements provide the most spatially localized representation of predicted RNA exposure, whereas progressively lower weights were assigned to broader local-window accessibility metrics. The weighting scheme was defined heuristically and was not derived from a fitted statistical model or experimentally calibrated against m6A occupancy. Accordingly, the composite score was used exclusively as a structure-informed prioritization metric and should not be interpreted as a probability of methylation or evidence that an individual DRACH motif is methylated.

During revision, the structural-analysis outputs were systematically audited and the composite accessibility score was recalculated from the six RNAplfold-derived accessibility components using the weighting scheme specified above. The previously stored normalized score was found to be invariant across observations; therefore, min–max normalization was reapplied directly to the recalculated raw composite scores. This correction affected normalization and candidate ranking but did not alter the underlying RNAfold/RNAplfold predictions or the six accessibility measurements. However, the previously stored normalized score was found to be invariant across observations, resulting in an erroneous assignment of all observations to the highest-priority category. The normalization step was therefore corrected by applying min–max normalization directly to the verified raw composite scores. This correction affected normalization and candidate ranking but not the underlying RNAfold/RNAplfold predictions or the six accessibility measurements.

The corrected normalized score ranged from 0 to 1. An operational high-accessibility subset was defined as the highest-ranking 10% of fragment-level observations. This threshold was used solely for descriptive prioritization and was not considered a biologically validated cutoff. Candidate observations within this subset are therefore referred to as high-accessibility DRACH candidates or structure-prioritized DRACH candidates, rather than m6A hotspots.

#### 2.14.6. Sensitivity Analysis of the Composite Accessibility Score

Because the relative weights assigned to the six accessibility components were investigator-defined, the robustness of the resulting structural prioritization was evaluated using complementary sensitivity analyses. First, composite scores and candidate rankings were recalculated using equal weighting of all six RNAplfold-derived accessibility metrics. Second, leave-one-component-out analyses were performed by sequentially excluding each accessibility metric and recalculating the composite score using the remaining components, with their original weights rescaled to sum to 1. Third, a Monte Carlo sensitivity analysis was performed using 100,000 alternative weight vectors sampled from a Dirichlet distribution centered on the original weighting configuration (0.35, 0.25, 0.10, 0.15, 0.10, and 0.05), with a concentration parameter of 50. Each sampled weight vector was constrained to sum to 1.

Robustness was evaluated by comparing each alternative ranking with the original composite-score ranking using Spearman rank correlation and by quantifying the number of observations from the original top-decile candidate subset that remained within the top decile. For the Monte Carlo analysis, the distribution of Spearman rank correlations and top-decile retention was summarized across simulations. These analyses were intended solely to determine whether structural prioritization was sensitive to reasonable variation in investigator-defined weights and were not interpreted as validation of the weighting scheme or as evidence of m6A modification.

### 2.15. Statistical Analysis

All statistical analyses were conducted in R (v4.5.2) and SPSS (v26), and significance thresholds were interpreted within an exploratory framework given the transcript-level resolution of the analysis. Continuous variables are presented as medians with interquartile ranges (IQR) and were compared between APS and control groups using the Mann–Whitney U test due to non-normal distributions. Global m6A abundance was compared between APS patients and controls using Welch’s two-sample *t*-test because homogeneity of variance was not supported (Levene’s test, *p* = 0.002). Other between-group molecular comparisons were performed using the Mann–Whitney U test as specified above. Categorical variables are shown as absolute frequencies and percentages and were compared using Fisher’s exact test. A two-sided *p*-value < 0.05 was considered statistically significant.

### 2.16. Reproducibility and Data Availability

All analyses were conducted using R (v4.5.2) and ViennaRNA tools (version 2.7.2). Complete scripts, intermediate files, and final outputs are available in the associated Zenodo repository. All computational steps, parameters, and dependencies are documented to ensure reproducibility. Additional experimental and computational details are provided in the Supplementary Methods.

## 3. Results

The results are organized as a sequence of complementary experimental and computational analyses. First, we evaluate BRD3OS expression and independently validate its dysregulation in APS. Second, we characterize global m6A abundance, selected m6A regulators, and inflammatory mediators as molecular features of the APS PBMC environment. Third, integrative network and RNA-structure analyses are used to generate hypotheses regarding the potential regulatory context of BRD3OS and to prioritize structurally accessible DRACH candidate regions for future experimental investigation. The experimental and computational findings are therefore presented as complementary but distinct levels of evidence.

### 3.1. Characteristics of the Study Population

Table 1 summarizes the demographic, clinical, and serological characteristics of the study population. The validation cohort comprised 35 patients with APS and 24 healthy controls. The median age of the overall population was 36.0 years (IQR, 26.8–47.2); APS patients had a median age of 39.0 years (IQR, 27.0–46.0), compared with 31.0 years (IQR, 26.5–48.0) among controls. The sex distribution was similar between groups, with women representing 71.4% of the APS group and 70.8% of the control group.

Among patients with available APS subtype information, 19 (57.6%) had primary APS and 14 (42.4%) had secondary APS. Obstetric involvement was documented in 10 patients (30.3%). Antiphospholipid antibody positivity was frequent, including lupus anticoagulant in 17 patients (70.8% of those with available data), anticardiolipin antibodies in 26 (78.8%), and anti-β2-glycoprotein I antibodies in 28 (84.8%).

The APS group also had a greater burden of cardiovascular and metabolic comorbidities than the healthy control group. Hypertension was present in 6 APS patients (18.2%), diabetes mellitus in 3 (9.1%), ischemic heart disease in 4 (12.1%), and cerebrovascular disease in 9 (27.3%), whereas none of these conditions were documented among controls.

Given these baseline differences, exploratory analyses were performed to assess whether BRD3OS expression was associated with available demographic or clinical characteristics. Although APS patients had a higher median age than controls (39.0 [IQR, 27.0–46.0] vs. 31.0 [IQR, 26.5–48.0] years), the age distributions did not differ significantly between groups (Mann–Whitney U test, *p* = 0.629). Moreover, BRD3OS expression was not significantly correlated with age across the complete validation cohort (Spearman ρ = −0.134, *p* = 0.386). No significant association was observed when APS patients (ρ = 0.133, *p* = 0.535) and controls (ρ = −0.230, *p* = 0.329) were examined separately.

Within the APS group, BRD3OS expression was not significantly associated with hypertension (*p* = 1.000), diabetes mellitus (*p* = 0.355), ischemic heart disease (*p* = 0.181), cerebrovascular disease (*p* = 0.664), systemic lupus erythematosus status (*p* = 0.562), APS subtype (primary versus secondary; *p* = 0.507), or obstetric involvement (*p* = 0.783). Collectively, these exploratory analyses did not identify measurable associations between BRD3OS expression and the available demographic or clinical variables. However, given the sample size and the exploratory nature of these analyses, residual confounding cannot be excluded. Medication exposure was not available in the recovered analytical dataset and therefore could not be evaluated.

### 3.2. Identification, Robustness Assessment, and Independent Validation of BRD3OS Dysregulation in APS

**Figure 1:**
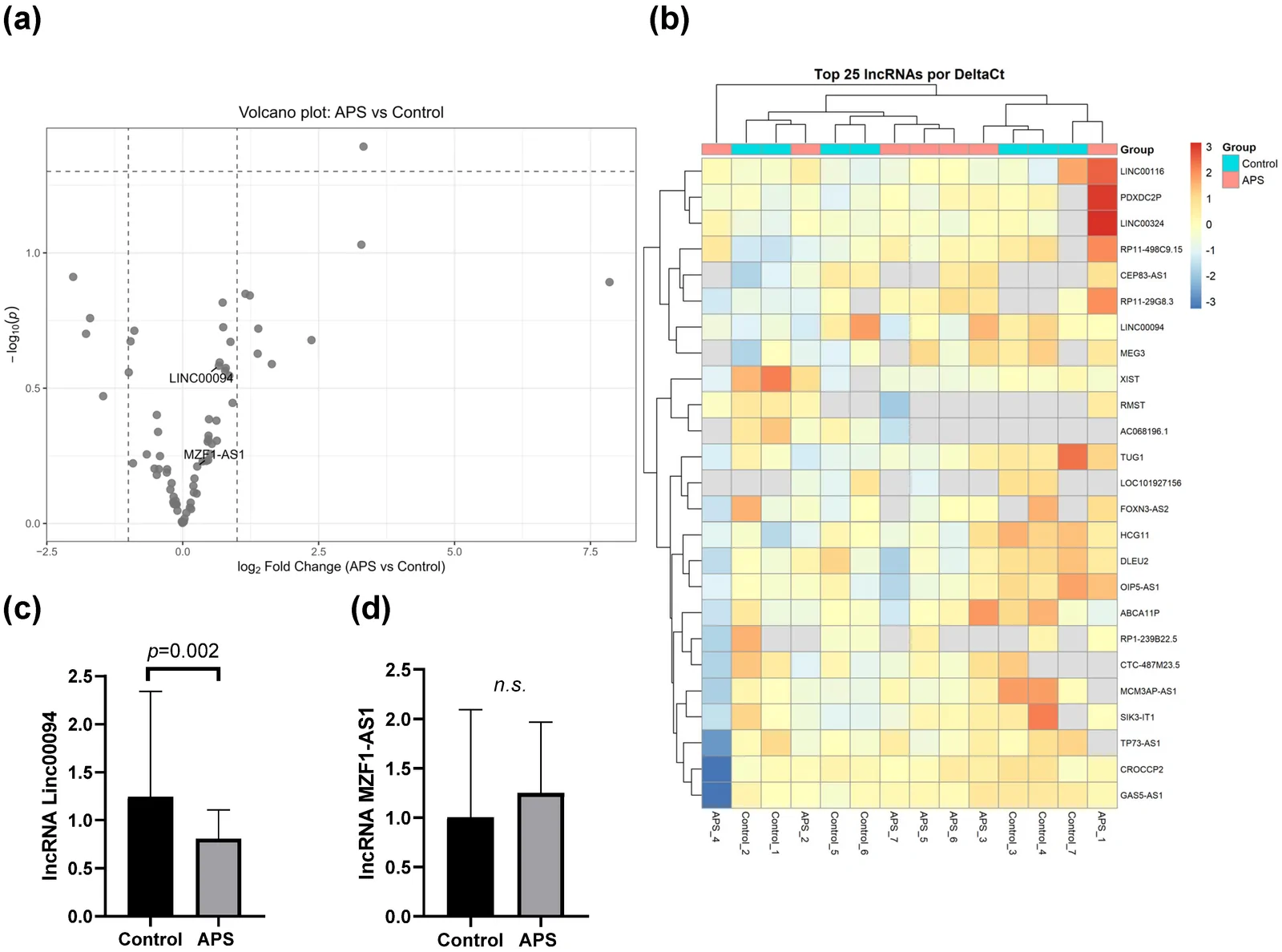
Discovery and independent validation of BRD3OS (LINC00094) dysregulation in antiphospholipid syndrome (APS). (**a**) Volcano plot showing exploratory differential-expression results for lncRNAs in PBMCs from patients with APS and healthy controls in the discovery cohort (*n* = 7 per group). No transcript remained statistically significant after multiple-testing correction. BRD3OS (LINC00094) and MZF1-AS1 are highlighted as candidates subsequently evaluated in the validation phase. Each point represents one analyzed lncRNA. The vertical dashed lines indicate log2 fold-change thresholds of −1 and +1, and the horizontal dashed line indicates the nominal p = 0.05 threshold. (**b**) Heatmap of the top 25 lncRNAs ranked according to ΔCt-based expression patterns, illustrating interindividual heterogeneity and partial clustering by study group. (**c**) Independent RT-qPCR validation of BRD3OS expression (APS, *n* = 35; controls, *n* = 24), showing significantly lower expression in APS (*p* = 0.002, Mann–Whitney U test). (**d**) Independent RT-qPCR evaluation of MZF1-AS1 in the same cohort, showing no significant between-group difference (n.s.).

An exploratory RT^2^ lncRNA PCR array was performed in PBMCs from 7 patients with APS and 7 healthy controls to identify lncRNAs for subsequent prioritization and independent validation. As shown in Figure 1a, the discovery analysis revealed modest and heterogeneous expression differences, and no transcript remained statistically significant after Benjamini–Hochberg correction for multiple testing. Accordingly, the discovery dataset was treated as a hypothesis-generating screen rather than as confirmatory evidence of differential expression.

Unsupervised clustering of the top 25 lncRNAs according to ΔCt-based expression patterns likewise showed substantial interindividual heterogeneity and only partial separation between APS and control samples (Figure 1b). Candidate selection was therefore based on the multi-parameter prioritization framework described in Section 2.7, which integrated detectability, expression variability, effect size, amplification characteristics, and exploratory statistical support rather than nominal statistical significance alone.

BRD3OS (LINC00094) showed stable prioritization across alternative weighting strategies. Under the original continuous weighting scheme, BRD3OS ranked 8th among the 69 evaluated candidates, whereas equal weighting placed it 6th. Across leave-one-component-out analyses, its position ranged from 4th to 11th. Specifically, BRD3OS ranked 8th after exclusion of detectability, 11th after exclusion of inverse variability, 8th after exclusion of effect size, 4th after exclusion of inverse mean Ct, and 11th after exclusion of exploratory statistical support. In the Monte Carlo sensitivity analysis comprising 100,000 perturbations of the weighting configuration, BRD3OS had a median rank of 8 (IQR, 7–9) and remained among the top 10 candidates in 91.1% of simulations. Thus, BRD3OS prioritization was generally stable across reasonable perturbations of the scoring framework, although its precise position varied according to the contribution of individual components. These analyses were used solely to evaluate ranking robustness and were not considered additional evidence of differential expression.

BRD3OS and MZF1-AS1 were subsequently evaluated by targeted RT-qPCR in the independent validation cohort comprising 35 patients with APS and 24 healthy controls. BRD3OS expression was significantly reduced in APS compared with controls (*p* = 0.002, Mann–Whitney U test; Figure 1c), independently supporting the dysregulation suggested by the exploratory discovery phase. In contrast, MZF1-AS1 did not differ significantly between groups (Figure 1d), illustrating that prioritization in the discovery screen did not necessarily translate into reproducible between-group differences in the independent cohort.

As an additional quality-control assessment for the validation analysis, the Cq distributions of the endogenous reference genes were examined directly in the validation cohort. RPLP0 Cq values were comparable between APS patients and controls (median, 18.95 vs. 18.87; *p* = 0.696), as were GAPDH Cq values (median, 20.07 vs. 20.23; *p* = 0.183). The combined mean Cq of the two reference genes also showed no significant between-group difference (*p* = 0.294). Moreover, RPLP0 and GAPDH Cq values were strongly correlated across samples (Spearman ρ = 0.803, *p* < 0.001), supporting their combined use for normalization of the targeted RT-qPCR measurements.

### 3.3. Global m6A Abundance, Selected m6A Regulators, and Inflammatory Mediators in APS

**Figure 2:**
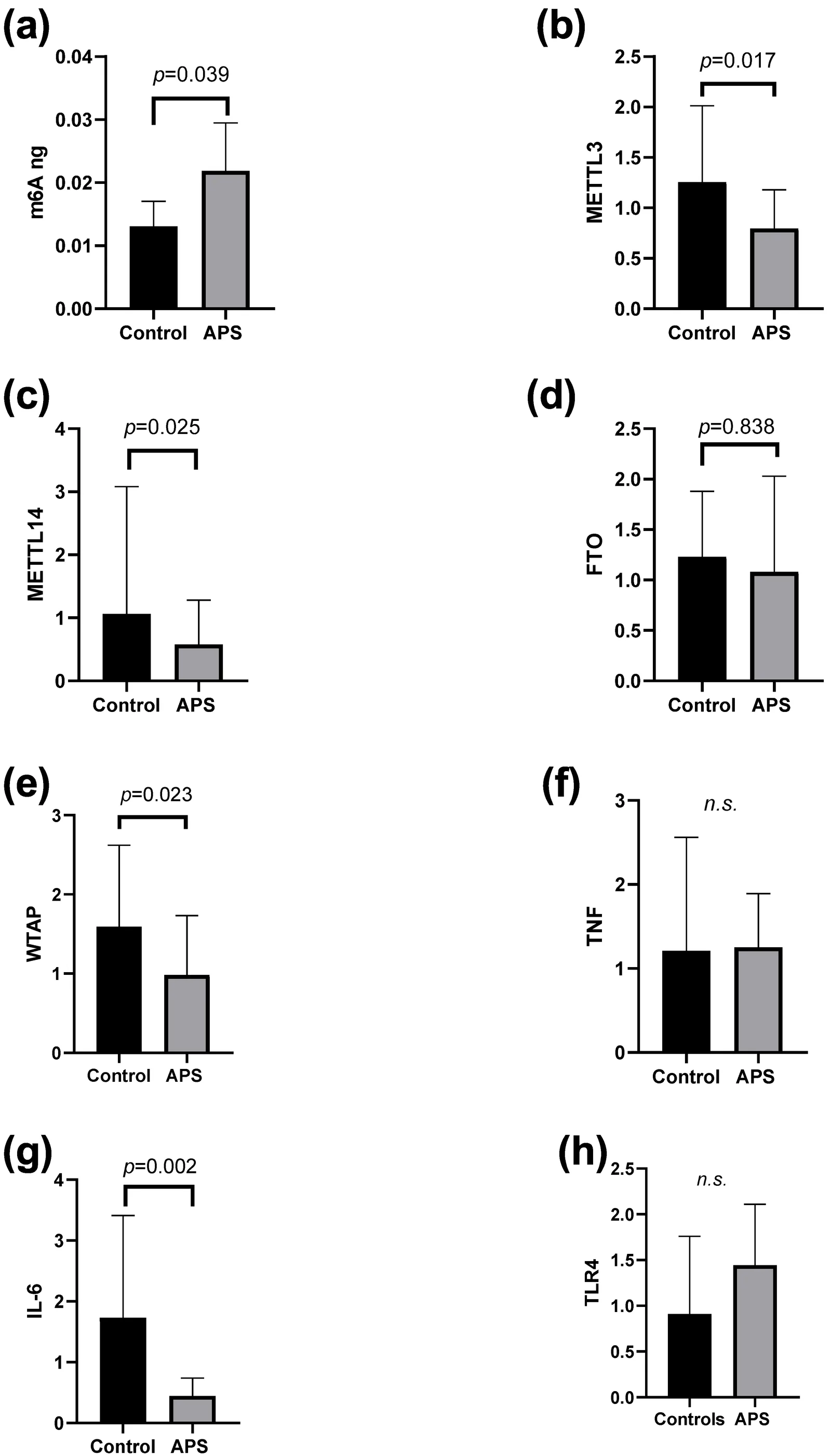
Global m6A abundance and expression of selected m6A regulators and inflammatory mediators in antiphospholipid syndrome (APS). (**a**) Global m6A abundance measured in total RNA from peripheral blood mononuclear cells (PBMCs), showing higher levels in APS patients than in controls (*p* = 0.039, Welch’s *t*-test). (**b**,**c**,**e**) Relative expression of the m6A methyltransferase components METTL3, METTL14, and WTAP, respectively, showing lower expression in APS (METTL3, *p* = 0.017; METTL14, *p* = 0.025; WTAP, *p* = 0.023). (**d**) Relative expression of the m6A demethylase FTO, showing no significant between-group difference (*p* = 0.838). (**f**–**h**) Relative expression of the inflammatory mediators TNF, IL6, and TLR4, respectively. IL6 expression was significantly lower in APS (*p* = 0.002), whereas TNF and TLR4 showed no significant between-group differences. Global m6A abundance in panel A was compared using Welch’s *t*-test because of unequal variances; RT-qPCR expression comparisons in panels B–H were performed using the Mann–Whitney U test. Error bars and summary statistics are presented according to the statistical approach used for each analysis. n.s., not significant.

To characterize the broader m6A-related molecular context in APS, global m6A abundance in total PBMC RNA was evaluated together with the expression of selected components of the m6A regulatory machinery. Global m6A abundance was higher in APS patients than in controls (mean, 0.0219 vs. 0.0131 ng; Welch’s *t*-test: t = −2.163, df = 27.309, *p* = 0.039; mean difference [control − APS], −0.00879 ng; 95% CI, −0.01712 to −0.00046; Figure 2a). This measurement reflects m6A abundance in the total RNA pool analyzed and cannot be attributed to BRD3OS or to any individual transcript.

Expression analysis of the selected m6A regulatory genes showed lower levels of the methyltransferase components METTL3 (*p* = 0.017; Figure 2b), METTL14 (*p* = 0.025; Figure 2c), and WTAP (*p* = 0.023; Figure 2e) in APS compared with controls. In contrast, expression of the demethylase FTO did not differ significantly between groups (*p* = 0.838; Figure 2d). Thus, increased global m6A abundance coexisted with reduced expression of the three measured methyltransferase components. Because the present study evaluated only a subset of the m6A regulatory machinery and did not assess enzymatic activity, transcript-specific methylation, or additional regulators such as ALKBH5, VIRMA, or METTL16, the basis of this discordant pattern cannot be determined from the present data.

Inflammatory mediators were evaluated in parallel to provide an exploratory molecular context for the PBMC expression profile. TNF expression did not differ significantly between APS patients and controls (Figure 2f), whereas IL6 expression was lower in APS (*p* = 0.002; Figure 2g). TLR4 expression likewise showed no significant between-group difference (Figure 2h). These inflammatory measurements were included as complementary markers of immune-related transcriptional status and were not used to infer a direct regulatory relationship with BRD3OS or global m6A abundance.

### 3.4. Integrative Bioinformatic Network Contextualizes BRD3OS Within APS- and m6A-Related Molecular Systems

**Figure 3:**
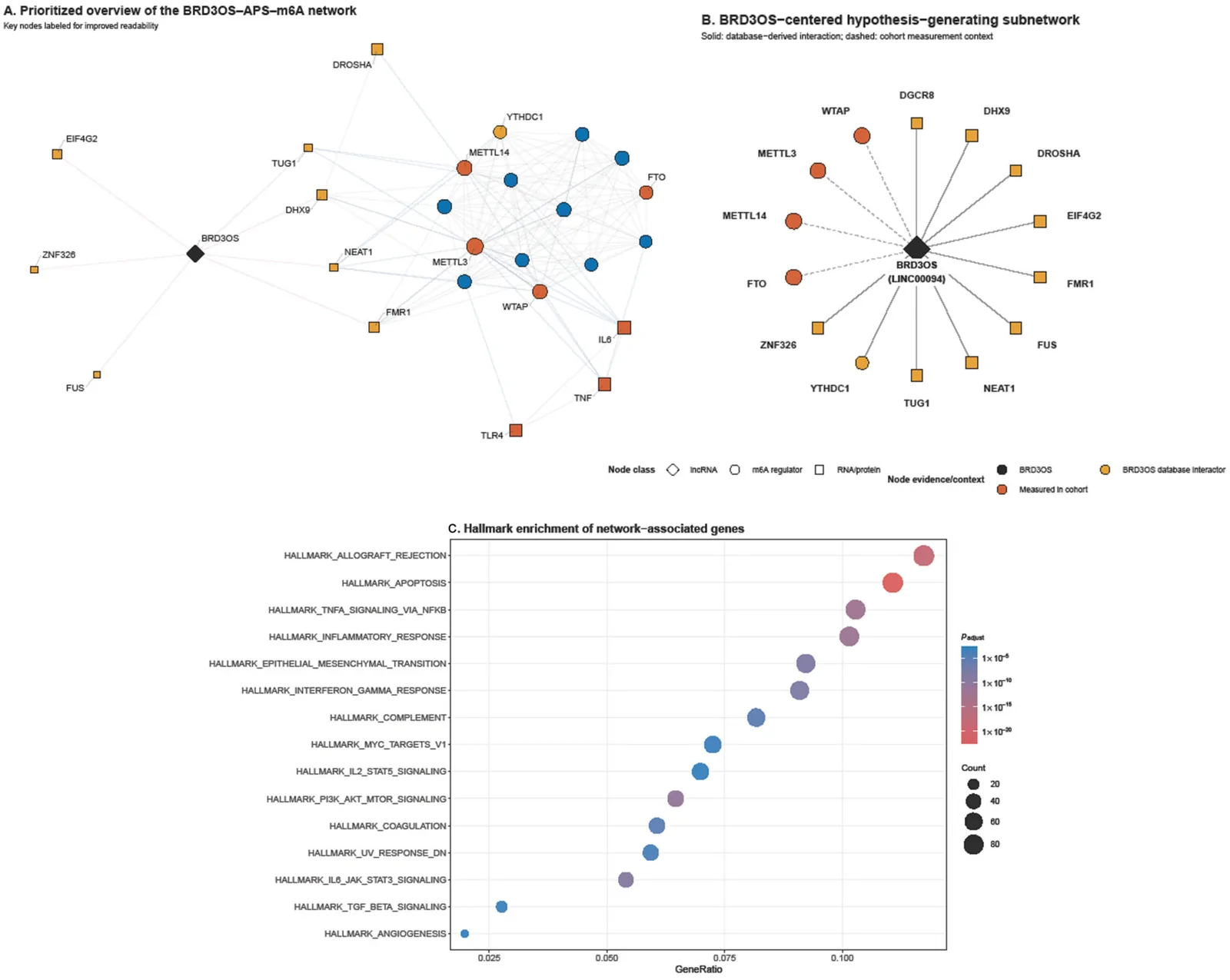
Integrative bioinformatic contextualization of BRD3OS within APS and m6A-related molecular systems. (**A**) Prioritized overview of the BRD3OS–APS–m6A network, representing a visualization-only reduction in the deposited integrative network to improve interpretability while retaining BRD3OS-centered, experimentally measured, m6A-related, and highly prioritized network components. Edges represent database-derived molecular relationships and are shown for contextualization rather than as experimentally validated interactions. (**B**) BRD3OS-centered hypothesis-generating subnetwork showing database-supported RNA–RNA and RNA–protein interactions together with selected molecules measured in the study cohort. Solid edges denote database-derived interactions, whereas dashed edges indicate cohort measurement context and should not be interpreted as demonstrated molecular interactions with BRD3OS. (**C**) Hallmark gene-set enrichment of network-associated genes. Dot size represents gene count, whereas color represents the Benjamini–Hochberg-adjusted *p*-value; node colors in panels (**A**,**B**) denote evidence/context categories as defined in the figure legend. Collectively, the network relationships and enrichment results shown in this figure provide bioinformatic and database-derived molecular context and should be interpreted as hypothesis-generating rather than as experimental evidence of BRD3OS-mediated regulation. Within this integrative network, BRD3OS occupied a peripheral but connected position relative to RNA-interacting molecules, APS-associated genes, and components of the m6A regulatory machinery (**A**). Functional node classification distinguished lncRNAs, protein-coding genes, and m6A-related writers, readers, and erasers, allowing the different information layers contributing to the network to be visualized. These relationships reflect computational integration of public interaction and disease-association resources and do not establish direct physical or functional interactions in APS PBMCs.

To explore the potential molecular context of BRD3OS in APS, an integrative bioinformatic network was constructed by combining APS-associated genes, predicted or database-supported BRD3OS interactions, m6A-related components, and protein–protein interaction data. The resulting filtered network is presented as a hypothesis-generating representation of database-derived molecular relationships rather than as an experimentally validated regulatory network (Figure 3A).

A focused BRD3OS subnetwork was subsequently extracted to visualize the database-supported interactions directly connected to BRD3OS after the predefined network-filtering procedure (Figure 3B). This subnetwork included RNA–RNA and RNA–protein relationships involving regulatory transcripts and RNA-binding proteins. Among the represented interactors were RNA-binding proteins implicated in post-transcriptional regulation, providing candidate molecular relationships for subsequent experimental investigation. Importantly, their inclusion in the network indicates database-supported association rather than experimental validation in the present cohort.

Functional enrichment analysis of the network-associated gene set identified overrepresented Hallmark gene sets related to inflammatory, immune, coagulation, and signaling processes (Figure 3C). Enrichment significance was evaluated using Benjamini–Hochberg-adjusted *p*-values. These results provide functional context for genes represented within the integrative network but do not demonstrate pathway activation, nor do they establish that the enriched processes are functionally regulated by BRD3OS in APS.

### 3.5. Structural Mapping of DRACH Motifs Across BRD3OS

**Figure 4:**
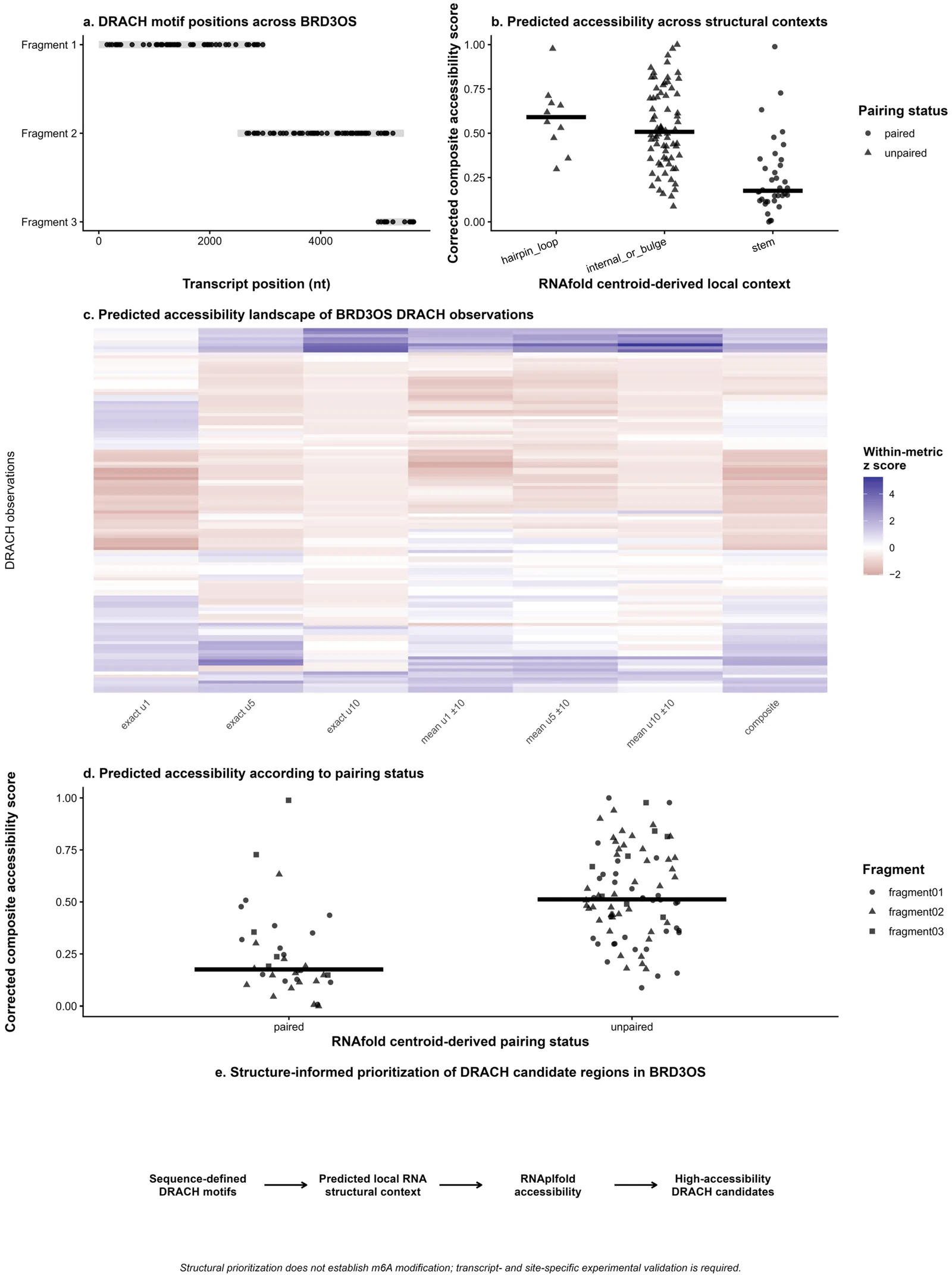
Structure-informed analysis and prioritization of DRACH motifs across the BRD3OS transcript. (**a**) Distribution of fragment-level DRACH observations across the three overlapping regions used for structural analysis of BRD3OS. A total of 120 fragment-level observations corresponding to 106 unique transcript positions were analyzed. (**b**) Corrected composite RNAplfold-derived accessibility scores according to RNAfold centroid-derived local structural context. Points represent individual fragment-level DRACH observations and shapes indicate predicted paired/unpaired status; horizontal lines indicate group medians. Accessibility differed among structural contexts (Kruskal–Wallis H = 36.335, *p* = 1.29 × 10^−8^). (**c**) Accessibility landscape across DRACH observations based on standardized RNAplfold-derived accessibility metrics and the corrected composite score. Values were standardized within each metric for visualization only and do not represent methylation probabilities. (**d**) Corrected composite accessibility according to predicted paired/unpaired status, with point shapes indicating transcript fragment. Unpaired observations exhibited greater predicted accessibility than paired observations (Wilcoxon W = 468, *p* = 2.29 × 10^−9^). (**e**) Conceptual representation of the computational prioritization framework, integrating sequence-defined DRACH motifs, predicted local RNA structure, and RNAplfold-derived accessibility to identify high-accessibility DRACH candidates. These candidates represent structure-prioritized regions and should not be interpreted as predicted or experimentally confirmed m6A modification sites. Transcript- and site-specific methylation mapping is required for validation.

Sequence-based screening identified multiple DRACH consensus motifs across the three overlapping fragments of the BRD3OS transcript (Figure 4a). At the fragment-analysis level, 120 DRACH observations were identified, comprising 49 sites in fragment 1, 57 in fragment 2, and 14 in fragment 3. Because adjacent fragments contained overlapping sequence intervals, this fragment-level count includes motifs represented independently in more than one fragment and therefore should not be interpreted as the number of unique transcript-level DRACH positions.

RNAfold centroid-based annotation classified 36 of the 120 fragment-level DRACH observations as paired and 84 as unpaired. According to local structural context, 10 were annotated as hairpin loops, 74 as internal/bulge regions, and 36 as stems. These classifications describe the predicted structural context of the analyzed DRACH observations under the computational folding framework and do not establish whether any individual motif is methylated in vivo.

The distribution map shown in Figure 4a therefore provides a sequence- and structure-based representation of candidate DRACH positions across BRD3OS. Because no formal null model was used to test spatial enrichment or clustering of DRACH motifs along the transcript, their observed distribution was not interpreted as statistically non-random or as evidence of preferential localization.

### 3.6. Predicted RNA Accessibility Varies Across DRACH Structural Contexts

RNAplfold analysis revealed heterogeneous predicted accessibility across BRD3OS DRACH observations (Figure 4b–d). Accessibility was evaluated using exact-site probabilities (u1, u5, and u10) together with corresponding local-window measurements calculated within ±10 nt of the central DRACH adenosine. The resulting profiles showed substantial site-to-site variation across the transcript, indicating that sequence-defined DRACH motifs occur within heterogeneous predicted structural environments (Figure 4c).

When DRACH observations were examined according to RNAfold centroid-derived structural annotations, unpaired positions showed higher exact accessibility than paired positions for each of the three evaluated exact-site metrics (exact_u1, *p* = 5.27 × 10^−9^; exact_u5, *p* = 5.51 × 10^−6^; exact_u10, *p* = 1.06 × 10^−3^). Consistent differences were also observed using the corrected composite accessibility score, which was higher among unpaired than paired observations (Wilcoxon rank-sum test, W = 468, *p* = 2.29 × 10^−9^; Figure 4d). Composite accessibility also differed across RNAfold centroid-derived local structural contexts (Kruskal–Wallis H = 36.335, *p* = 1.29 × 10^−8^; Figure 4b).

The operational top decile of the corrected composite score comprised 12 fragment-level observations corresponding to 10 unique transcript positions. Eleven of the 12 observations (91.7%) were classified as unpaired, and 10 (83.3%) occurred within internal loop/bulge contexts. These findings indicate that the highest predicted accessibility values were predominantly associated with locally unpaired structural environments. However, because these classifications and accessibility estimates are computational predictions, they should not be interpreted as experimental confirmation of motif exposure in vivo or evidence of m6A deposition.

Potential effects introduced by fragment-wise folding were evaluated separately through the overlap-consistency and weighting-sensitivity analyses described in Section 3.7.

### 3.7. Fragment-Boundary Consistency and Robustness of Structural Prioritization

Fourteen DRACH positions were independently represented in the overlapping regions between adjacent BRD3OS fragments, enabling direct assessment of potential boundary effects introduced by fragment-wise folding. RNAplfold-derived quantitative accessibility estimates showed high cross-fragment concordance. The corrected composite accessibility score was strongly correlated between duplicate representations (Spearman ρ = 0.969; Pearson r = 0.994), indicating that quantitative accessibility estimates were largely preserved when the same transcript position was evaluated within independently folded overlapping fragments.

Discrete RNAfold-derived structural classifications showed greater sensitivity to fragment context. Pairing-status classification agreed for 11 of 14 duplicated positions (78.6%), whereas local structural-context classification agreed for 10 of 14 positions (71.4%). Thus, quantitative accessibility estimates were comparatively robust across fragment boundaries, whereas categorical structural annotations were more sensitive to the surrounding folding context. The complete site-level fragment-overlap consistency analysis is provided in Supplementary Data S2.

Sensitivity analyses were subsequently used to evaluate dependence of the composite accessibility ranking on the investigator-defined weighting scheme. Recalculation using equal weights produced a ranking strongly correlated with the original composite ranking (Spearman ρ = 0.974), with 7 of the 12 original top-decile observations remaining within the top decile. Leave-one-component-out analyses similarly showed high overall rank concordance, with Spearman correlations ranging from 0.786 to approximately 1.000 and retention of 7–12 of the 12 original top-decile observations. The greatest change occurred after exclusion of exact_u1 (ρ = 0.786; 7/12 retained), the component assigned the greatest weight in the original formulation.

Across 100,000 Dirichlet-perturbed weighting configurations, alternative rankings remained highly concordant with the original ranking, with a median Spearman correlation of 0.997 (IQR, 0.995–0.999). The median number of original top-decile observations retained was 11 of 12 (IQR, 11–12). The complete structural accessibility-score sensitivity analysis is provided in Supplementary Data S3 and summarized in Supplementary Table S2.

## 4. Discussion

In this study, we identified BRD3OS (LINC00094) as a reproducibly downregulated long non-coding RNA in peripheral blood mononuclear cells (PBMCs) from patients with antiphospholipid syndrome (APS). This finding emerged from an exploratory discovery screen and was subsequently supported by targeted RT-qPCR in an independent validation cohort. In parallel, APS was characterized by increased global m6A abundance in total PBMC RNA, reduced expression of the selected methyltransferase components METTL3, METTL14, and WTAP, and altered expression of selected inflammatory mediators. Integrative network and RNA-structure analyses further provided a computational framework for contextualizing BRD3OS and prioritizing structurally accessible DRACH motifs for future experimental investigation. Importantly, these findings represent complementary but distinct levels of evidence: the present study demonstrates altered BRD3OS expression and global m6A-related molecular features in APS, whereas the network and structural analyses remain hypothesis-generating and do not establish transcript-specific methylation or a causal relationship between these observations.

The discovery phase did not identify lncRNAs that remained statistically significant after correction for multiple testing, consistent with the limited sample size and the exploratory nature of the initial screen. Rather than interpreting nominally significant signals as definitive, candidate selection was therefore approached as a prioritization problem integrating detectability, expression variability, effect size, amplification characteristics, and exploratory statistical support. Importantly, sensitivity analyses showed that BRD3OS prioritization remained stable under equal weighting, leave-one-component-out models, and extensive perturbation of the original weighting configuration. The subsequent independent validation of BRD3OS downregulation provides the principal experimental support for its association with APS and reduces dependence of the central finding on the initial discovery-ranking procedure. More broadly, this strategy illustrates the potential value of separating candidate prioritization from confirmatory inference in small exploratory transcriptomic datasets.

The biological significance of BRD3OS downregulation remains to be established. lncRNAs can participate in gene regulation through diverse and highly context-dependent mechanisms, including interactions with chromatin-associated factors, RNA-binding proteins, other RNA species, and signaling pathways. Recent synthesis of lncRNA-mediated regulation in pharmacological and immune-related contexts similarly emphasizes that lncRNAs can act as context-dependent regulatory modifiers while also highlighting the substantial evidentiary gap that often separates expression associations and computational predictions from experimentally demonstrated function [18]. In this context, the reproducible reduction in BRD3OS observed here identifies it as a candidate APS-associated transcript but does not establish whether its altered expression contributes to, results from, or is independent of the thrombo-inflammatory processes underlying APS. Functional perturbation studies will therefore be necessary to determine whether BRD3OS influences disease-relevant cellular phenotypes.

A second finding was the coexistence of increased global m6A abundance with reduced expression of METTL3, METTL14, and WTAP. This pattern should be interpreted cautiously. Global m6A abundance represents the aggregate methylation signal of the RNA pool analyzed and is determined by multiple processes beyond steady-state expression of the three measured methyltransferase components. The present study did not comprehensively characterize the m6A regulatory machinery: other relevant regulators, including ALKBH5, VIRMA, METTL16, additional readers and associated factors, were not measured, nor were protein abundance or enzymatic activity evaluated. Consequently, the observed pattern cannot establish compensatory regulation, altered substrate selectivity, redistribution of methylation, or any other specific mechanism. Rather, our results indicate that global RNA methylation abundance and expression of the selected m6A regulators are concurrently altered in APS and that their relationship remains mechanistically unresolved [19,20].

This distinction is particularly important when considering BRD3OS. Global m6A was quantified in total PBMC RNA and therefore cannot provide information regarding the methylation state of an individual transcript. Accordingly, the increased global m6A signal cannot be attributed to BRD3OS, and the present data do not demonstrate that BRD3OS downregulation is mediated by m6A. Similarly, the reduced expression of METTL3, METTL14, and WTAP does not establish altered methyltransferase activity at BRD3OS. The experimental observations should therefore be considered evidence of a broader m6A-related molecular alteration in APS rather than evidence of transcript-specific epitranscriptomic regulation.

The integrative network analysis provides an additional, but explicitly computational, level of context. Database-derived relationships positioned BRD3OS within an RNA-centered network containing RNA-binding proteins and molecules associated with APS and m6A-related regulation [7,21,22,23]. Interactions involving proteins such as FMR1, FUS, DHX9, and EIF4G2 are compatible with the broader capacity of lncRNAs to participate in ribonucleoprotein and post-transcriptional regulatory systems [7,24]. However, these relationships were derived from public interaction resources and were not experimentally demonstrated in APS PBMCs. Likewise, pathway enrichment reflects the functional composition of the network-derived gene set rather than direct evidence that BRD3OS regulates those pathways. Figure 3 should therefore be interpreted as a hypothesis-generating map that prioritizes potential molecular relationships for subsequent experimental interrogation.

The structural analysis further extends this hypothesis-generating framework by showing that DRACH motifs within BRD3OS occur in heterogeneous predicted RNA environments and exhibit markedly different RNAplfold-derived accessibility. Importantly, predicted accessibility was significantly greater among unpaired than paired observations, and the highest-accessibility subset was predominantly composed of unpaired motifs located within internal loop/bulge contexts. Similar differences were observed for the individual RNAplfold accessibility measurements, indicating that this relationship was not solely generated by the composite scoring procedure. These findings are consistent with the broader principle that local RNA structure can modulate nucleotide exposure and interactions with RNA-binding proteins or RNA-modifying machinery [25]. Foundational transcriptome-wide m6A mapping studies have also established recurrent sequence and positional features of m6A deposition [26,27], while RNAplfold and the ViennaRNA framework provide established computational approaches for estimating local accessibility and RNA secondary structure [28,29]. They do not, however, demonstrate that structurally accessible BRD3OS motifs are methylated or that accessibility determines m6A deposition.

Fragment-boundary analysis provided an additional assessment of the computational framework. Quantitative accessibility estimates for DRACH positions independently represented in overlapping fragments were highly concordant, including a Spearman correlation of 0.969 and Pearson correlation of 0.994 for the composite accessibility score. In contrast, discrete pairing and structural-context annotations showed lower agreement across fragment boundaries. This distinction suggests that probabilistic accessibility estimates are relatively robust to the fragmentation strategy, whereas categorical secondary-structure assignments may be more sensitive to the sequence context supplied to the folding algorithm. Accordingly, site-level structural classifications should be interpreted cautiously, particularly near fragment boundaries. 

Sensitivity analysis further supported the numerical robustness of the composite prioritization framework. Across 100,000 alternative weighting configurations, rankings remained highly concordant with the original composite ranking, with a median Spearman correlation of 0.997 (IQR, 0.995–0.999), while a median of 11 of the 12 original top-decile observations were retained (IQR, 11–12). Nevertheless, because the weighting scheme was investigator-defined and was not experimentally calibrated against m6A occupancy, these analyses should be interpreted as evidence of computational stability rather than biological validation. Accordingly, the highest-ranked motifs are considered structure-prioritized candidates for subsequent investigation rather than predicted m6A hotspots.

Recent methodological developments provide useful context for this distinction. Choudhari et al. developed the transcript-aware m6A-FINDiT framework, which incorporates RNA secondary structure into the identification and prioritization of potential m6A motifs and complements computational prediction with motif-specific experimental assessment [30]. Their approach illustrates an important distinction that is directly relevant to the present study: incorporating structural information can refine sequence-based candidate selection, but demonstration of methylation requires an independent transcript- or site-specific experimental layer. Our RNAfold/RNAplfold framework extends sequence-based DRACH screening by incorporating probabilistic local accessibility, boundary-consistency assessment, and sensitivity analyses of the composite prioritization scheme. However, unlike an experimentally validated methylation-mapping workflow, it does not establish that any prioritized BRD3OS motif is methylated in APS.

This distinction also applies to the presence of the m6A reader YTHDC1 within the exploratory network [31]. Although such a relationship provides a biologically interesting candidate connection between BRD3OS and m6A-associated RNA regulation, its presence in a database-derived network does not demonstrate physical interaction, reader occupancy, or functional dependence in APS. The convergence of BRD3OS downregulation, altered global m6A-related measurements, predicted network relationships, and structurally accessible DRACH candidates therefore provides a rationale for targeted follow-up experiments, but should not be interpreted as evidence that these observations constitute a single demonstrated molecular pathway.

The inflammatory measurements provide additional context but similarly warrant cautious interpretation. IL6 expression was reduced in APS PBMCs, whereas TNF and TLR4 did not show significant between-group differences. These markers were included to characterize selected components of the inflammatory transcriptional environment rather than to define a BRD3OS-dependent inflammatory mechanism. The absence of coordinated changes across all three markers also illustrates the complexity of inferring inflammatory activity from bulk PBMC transcript measurements. Whether BRD3OS is related to specific immune signaling pathways will require cell-resolved and perturbation-based approaches. More broadly, previous proof-of-concept work has identified lncRNA dysregulation in APS, supporting continued investigation of non-coding RNA phenotypes in this disease [32].

Several limitations should therefore be considered. First, the discovery cohort was small, and no lncRNA survived multiple-testing correction. Although candidate-ranking sensitivity analyses and independent validation substantially strengthen the evidence for BRD3OS downregulation, the discovery phase remains exploratory. Second, PBMCs were analyzed as an unsorted bulk population. Differences in monocyte, T-cell, B-cell, or other leukocyte proportions may therefore contribute to the observed expression patterns, and the cellular origin of BRD3OS downregulation cannot be determined from the present data. Third, APS patients differed from healthy controls in age and comorbidity burden. Exploratory analyses did not identify significant associations between BRD3OS expression and age or the available clinical characteristics; however, the sample size limits the ability to exclude residual confounding. Medication exposure was not available in the analytical dataset and could not be evaluated, which is particularly relevant given the potential effects of anticoagulant, immunomodulatory, or other therapies on cellular transcriptional states.

Fourth, the m6A regulatory system was only partially characterized. METTL3, METTL14, WTAP, and FTO do not encompass the full complement of writers, erasers, readers, cofactors, or regulatory processes governing RNA methylation, and mRNA expression does not necessarily reflect protein abundance or enzymatic activity. Fifth, global m6A quantification lacks transcript-level resolution and cannot establish the methylation status of BRD3OS. Sixth, DRACH identification, RNAfold structural annotation, and RNAplfold accessibility estimates are computational predictions. Although the overlapping-fragment and weighting-sensitivity analyses provide internal assessments of computational robustness, they cannot reproduce the full complexity of RNA folding, RNA–protein interactions, subcellular localization, or dynamic structural remodeling in vivo. Finally, neither transcript-specific m6A mapping nor BRD3OS gain- or loss-of-function experiments were performed; therefore, no causal relationship can be established between BRD3OS, m6A regulation, and thrombo-inflammatory phenotypes.

These limitations define a focused experimental path forward. Transcript-specific approaches such as MeRIP-qPCR could first determine whether BRD3OS is enriched in the m6A-associated RNA fraction, while higher-resolution approaches such as miCLIP [33], or direct RNA sequencing-based strategies [34,35] could evaluate the structurally prioritized DRACH candidates. Site-directed perturbation could subsequently test whether individual candidate adenosines influence BRD3OS stability, localization, or molecular interactions. In parallel, BRD3OS gain- and loss-of-function experiments in relevant immune-cell populations will be required to determine whether the transcript contributes to inflammatory, endothelial, platelet-related, or other APS-relevant phenotypes. Cell-subset-resolved analyses would further help distinguish genuine transcriptional regulation from differences in PBMC composition.

## 5. Conclusions

BRD3OS (LINC00094) was reproducibly downregulated in PBMCs from patients with APS following independent validation of the initial exploratory finding. APS patients also exhibited increased global m6A abundance in total PBMC RNA together with reduced expression of the selected methyltransferase components METTL3, METTL14, and WTAP; however, these observations cannot be attributed specifically to BRD3OS. Integrative bioinformatic analyses provided complementary, hypothesis-generating evidence by placing BRD3OS within database-supported regulatory networks and identifying structure-prioritized high-accessibility DRACH candidate regions. These computational findings do not demonstrate m6A modification of BRD3OS or establish a causal role for BRD3OS in APS. Rather, the study defines a reproducible BRD3OS expression phenotype and provides a structurally informed framework for future transcript- and site-specific experimental investigation of its potential epitranscriptomic regulation.

## Figures and Tables

**Table 1:** Main characteristics of the study population.

Variable	Total (*n* = 59)	Control (*n* = 24)	APS (*n* = 35)
Age, median (IQR)	36.0 (26.8–47.2)	31.0 (26.5–48.0)	39.0 (27.0–46.0)
**Sex**			
Male	17 (28.8%)	7 (29.2%)	10 (28.6%)
Female	42 (71.2%)	17 (70.8%)	25 (71.4%)
**APS type**			
Primary APS	–	–	19 (57.6%)
Secondary APS	–	–	14 (42.4%)
**Obstetric involvement**			
Non-obstetric	–	–	23 (69.7%)
Obstetric	–	–	10 (30.3%)
**Lupus anticoagulant**			
Negative	–	–	7 (29.2%)
Positive	–	–	17 (70.8%)
**Anticardiolipin antibodies**			
Negative	–	–	7 (21.2%)
Positive	–	–	26 (78.8%)
**Anti-β2-glycoprotein I antibodies**			
Negative	–	–	5 (15.2%)
Positive	–	–	28 (84.8%)
**Hypertension**			
No	–	23 (100.0%)	27 (81.8%)
Yes	–	–	6 (18.2%)
**Diabetes**			
No	–	23 (100.0%)	30 (90.9%)
Yes	–	–	3 (9.1%)
**Ischemic heart disease**			
No	–	23 (100.0%)	29 (87.9%)
Yes	–	–	4 (12.1%)
**Cerebrovascular disease**			
No	–	23 (100.0%)	24 (72.7%)
Yes	–	–	9 (27.3%)

Data are shown as median (interquartile range) for continuous variables and as absolute frequency (percentage) for categorical variables. APS, antiphospholipid syndrome.

## Data Availability

The data and computational resources supporting the findings of this study are publicly available in the associated Zenodo repository at https://doi.org/10.5281/zenodo.19907750. The repository includes the raw and processed discovery RT-qPCR datasets; sample metadata and differential-expression outputs; complete candidate-prioritization and sensitivity-analysis data; source data and computational outputs underlying the BRD3OS-centered bioinformatic network; source data and scripts used to generate the revised Figure 3 and Figure 4; RNAInter-, CTD-, DisGeNET-, and RM2Target-derived analysis tables; BRD3OS transcript sequences and DRACH motif coordinates; RNAfold and RNAplfold outputs; fragment-overlap consistency results; structural accessibility-score sensitivity analyses; and the analysis scripts and computational instructions required to reproduce the principal bioinformatic analyses. The Supplementary Data and Tables accompanying the revised manuscript are also included in the repository to facilitate reproducibility and independent inspection of the reported analyses. De-identified aggregate clinical and expression data supporting the validation analyses are provided where permitted. Individual-level clinical information and potentially identifying participant data are not publicly shared because of ethical and privacy restrictions.

## Supplementary Materials

The following supporting information can be downloaded at: https://www.mdpi.com/article/10.3390/biom16091263/s1, Supplementary Methods, providing additional experimental and computational details, including primer information, software specifications, and analysis parameters; Supplementary Data S1, complete multi-parameter prioritization and sensitivity analysis of the 69 lncRNA candidates evaluated in the discovery cohort; Supplementary Table S1, summary of BRD3OS (LINC00094) candidate-prioritization robustness across the original, equal-weight, leave-one-component-out, and Monte Carlo weighting schemes; Supplementary Data S2, site-level fragment-overlap consistency analysis for BRD3OS DRACH positions independently represented in adjacent overlapping transcript fragments; Supplementary Data S3, complete sensitivity analysis of the BRD3OS structural accessibility composite score under alternative weighting configurations; and Supplementary Table S2, summary of structural accessibility-ranking robustness under equal-weight, leave-one-component-out, and Monte Carlo sensitivity analyses.

## Abbreviations

The following abbreviations are used in this manuscript:

Abbreviation	Definition
APS	Antiphospholipid Syndrome
PBMC	Peripheral Blood Mononuclear Cell
lncRNA	Long Non-Coding RNA
BRD3OS	Bromodomain Containing 3 Opposite Strand (LINC00094)
LINC00094	Long Intergenic Non-Protein Coding RNA 94
m6A	N6-methyladenosine
DRACH	Degenerate RNA consensus motif (D = A/G/U, R = A/G, H = A/C/U) associated with m6A methylation
RNAfold	ViennaRNA algorithm for minimum free-energy RNA secondary structure prediction
RNAplfold	ViennaRNA algorithm for local RNA accessibility prediction
RNAInter	RNA Interaction Database
STRING	Search Tool for the Retrieval of Interacting Genes/Proteins
CTD	Comparative Toxicogenomics Database
DisGeNET	Disease Gene Network Database
RM2Target	RNA Modification Targets Database
GO	Gene Ontology
KEGG	Kyoto Encyclopedia of Genes and Genomes
MSigDB	Molecular Signatures Database
PPI	Protein–Protein Interaction
RT–qPCR	Reverse Transcription Quantitative Polymerase Chain Reaction
RT^2^ PCR Array	Reverse Transcription Squared Polymerase Chain Reaction Array (QIAGEN)
Ct	Cycle Threshold
Cq	Quantification Cycle
ΔCt	Delta Cycle Threshold
ΔΔCt	Delta Delta Cycle Threshold
FDR	False Discovery Rate
BH	Benjamini–Hochberg
IQR	Interquartile Range
PBS	Phosphate-Buffered Saline
EDTA	Ethylenediaminetetraacetic Acid
cDNA	Complementary DNA
qPCR	Quantitative Polymerase Chain Reaction
RBP	RNA-Binding Protein
HGNC	HUGO Gene Nomenclature Committee
SLE	Systemic Lupus Erythematosus
IL6	Interleukin-6
TNF	Tumor Necrosis Factor
TLR4	Toll-Like Receptor 4
METTL3	Methyltransferase Like 3
METTL14	Methyltransferase Like 14
WTAP	Wilms Tumor 1-Associated Protein
FTO	Fat Mass and Obesity-Associated Protein
ALKBH5	AlkB Homolog 5, RNA Demethylase
YTHDC1	YTH Domain Containing 1
FMR1	Fragile X Messenger Ribonucleoprotein 1
FUS	Fused in Sarcoma
DHX9	DExH-Box Helicase 9
EIF4G2	Eukaryotic Translation Initiation Factor 4 Gamma 2
