# Supplementary material for: BRD3OS Dysregulation in Antiphospholipid Syndrome: Integrative Network and RNA Structural Analysis of m6A-Related Candidate Regions
Source: Biomolecules. 2026 Sep 1;16(9):1263. doi: 10.3390/biom16091263 (PMC13604529; doi:10.3390/biom16091263)
Supplement: Supplementary file 1 [file biomolecules-16-01263-s001.zip › biomolecules-4430342-supplementary.pdf]

Supplementary Materials

BRD3OS Dysregulation in Antiphospholipid Syndrome: Integrative Network and RNA Structural Analysis of m6A-Related Candidate Regions

This document consolidates the supplementary methodological information, datasets, and summary tables associated with the manuscript. Supplementary identifiers correspond to those cited in the main text.

Data and code availability. Underlying computational outputs, analysis scripts, and supporting data used to generate these supplementary materials are publicly archived in Zenodo (DOI: 10.5281/zenodo.19907750).

Contents

Supplementary Methods. Additional experimental and computational details, software specifications, analysis parameters, and variable definitions.

Supplementary Data S1. Complete multi-parameter prioritization and sensitivity analysis of the 69 lncRNA candidates evaluated in the discovery cohort.

Supplementary Table S1. Robustness of BRD3OS (LINC00094) candidate prioritization across alternative weighting schemes.

Supplementary Data S2. Fragment-overlap consistency analysis of BRD3OS DRACH candidate positions.

Supplementary Data S3. Complete site-level structural accessibility and composite-score sensitivity analysis of BRD3OS DRACH candidates.

Supplementary Table S2. Structural accessibility-ranking robustness under equal-weight, leave-one-component-out, and Monte Carlo sensitivity analyses.

Supplementary Methods

S1. Primer design and targeted RT-qPCR

Primer pairs used for targeted RT-qPCR were designed using NCBI Primer-BLAST (National Center for Biotechnology Information, Bethesda, MD, USA; Primer3 version 2.5.0), with preference for exon–exon junction-spanning designs to minimize genomic DNA amplification, and were synthesized by Integrated DNA Technologies (Coralville, IA, USA). Relative expression was calculated using the $2^{-\Delta\Delta Ct}$ method as described in the main Methods. The complete experimental context, reaction chemistry, and normalization strategy are reported in the main manuscript.

S2. Discovery-cohort candidate prioritization

Candidate prioritization was used only to select lncRNAs for independent validation and was not treated as confirmatory evidence of differential expression. Five normalized components were integrated: detectability (weight 0.30), inverse variability (0.20), effect size (0.20), inverse mean Ct (0.20), and nominal statistical support (0.10). Robustness was evaluated using equal weighting, leave-one-component-out analyses, and 100,000 Monte Carlo perturbations of the weighting vector. Complete candidate-level outputs are reported in Supplementary Data S1, with BRD3OS-specific results summarized in Supplementary Table S1.

S3. BRD3OS DRACH structural accessibility analysis

DRACH candidate positions were evaluated using RNAfold-derived structural annotations and RNAplfold-derived local accessibility estimates. The six accessibility components were exact_u1, exact_u5, exact_u10, mean_u1_pm10, mean_u5_pm10, and mean_u10_pm10. The final raw composite accessibility score was calculated as:

Composite score = 0.35(exact_u1) + 0.25(exact_u5) + 0.10(exact_u10) + 0.15(mean_u1_pm10) + 0.10(mean_u5_pm10) + 0.05(mean_u10_pm10)

The weighting scheme was heuristic and exploratory rather than statistically fitted or experimentally calibrated. The composite score was therefore used only for structure-informed prioritization and should not be interpreted as a probability of m6A deposition. Raw composite scores were min–max normalized across the final fragment-level observations. The top decile comprised the 12 highest-ranked fragment-level observations (10% of $n = 120$).

S4. Structural-score sensitivity analysis

Robustness of the final structural ranking was assessed by (i) equal weighting of the six accessibility components; (ii) six leave-one-component-out analyses, with the remaining weights renormalized to sum to 1; and (iii) 100,000 Monte Carlo weight perturbations sampled from a Dirichlet distribution centered on the final weight vector (0.35, 0.25, 0.10, 0.15, 0.10, 0.05) with concentration parameter 50. Rank stability was quantified using Spearman correlation with the final ranking and retention of the original top-decile set. The complete site-level results are reported in Supplementary Data S3 and summary results in Supplementary Table S2.

S5. Overlapping-fragment consistency

BRD3OS positions independently represented in adjacent overlapping transcript fragments were retained as separate fragment-level observations to assess boundary sensitivity. For duplicated full-transcript coordinates, paired/unpaired status, local structural context, RNAplfold accessibility estimates, and final composite scores were compared across fragment representations. Consequently, the same full-transcript coordinate may intentionally occur more than once in the structural dataset. The complete site-level comparison is provided in Supplementary Data S2.

S6. Software and computational reproducibility

Statistical and computational analyses were performed in R version 4.5.2 (R Foundation for Statistical Computing, Vienna, Austria). RNA secondary-structure and local-accessibility analyses used RNAfold and RNAplfold from the ViennaRNA software suite. Exact ViennaRNA executable versions were not recoverable from the archived provenance records available for this consolidated supplement; therefore, no version number is inferred here. The archived Zenodo repository contains the analysis scripts, structural outputs, and available session-information instructions required to audit the computational workflow.

S7. Variable definitions for supplementary datasets

Variable	Definition
detectability_raw	Observed discovery-cohort detection proportion before normalization.
variability_raw	Raw expression-variability measure used for candidate

	prioritization.
effect_size_raw	Raw magnitude of the discovery-phase expression effect used for prioritization.
mean_ct_raw	Raw mean Ct measure used in the prioritization framework.
p_value_raw	Nominal discovery-phase p value; used only as a prioritization component.
composite_score	Weighted five-component lncRNA candidate-prioritization score.
MC	Monte Carlo.
LOO	Leave-one-component-out.
Q1 / Q3	First and third quartiles, respectively.
a_pos_fragment	Adenosine coordinate relative to the analyzed fragment.
a_pos_full	Corresponding adenosine coordinate in the full BRD3OS transcript coordinate system used by the pipeline.
paired_status	Discrete RNAfold-derived paired/unpaired classification.
local_context	Discrete RNAfold-derived local structural-context annotation.
exact_u1 / exact_u5 / exact_u10	RNAplfold-derived accessibility metrics for window sizes u = 1, 5, and 10, respectively.
mean_u1_pm10 / mean_u5_pm10 / mean_u10_pm10	Mean local RNAplfold accessibility summaries within the ±10-nt neighborhood for the corresponding u values.
final_composite_raw	Weighted structural accessibility composite score before min–max normalization.
final_composite_normalized	Min–max normalized final structural accessibility composite score.
final_top_decile	Indicator that the fragment-level observation belongs to the 12 highest-ranked final observations.

Supplementary Data S1. Complete multi-parameter prioritization and sensitivity analysis of the 69 lncRNA candidates evaluated in the discovery cohort.

gene	detectability_raw	variability_raw	effect_size_raw	mean_ct_raw	p_value_raw	detectability	inverse_variability	effect_size	inverse_mean_ct	stat_support	composite_score	rank_original	equal_score	rank_equal	rank_without_detectability	rank_without_inverse_variability	rank_without_effect_size	rank_without_inverse_mean_ct	rank_without_stat_support	MC_median_rank	MC_Q1_rank	MC_Q3_rank	MC_top10_probability
RMST	0.428571	8.22968	7.8505	39.0333	0.128264	0	0	1	0.10021	0.639867	0.284029	68	0.348015	66	46	60	69	67	69	68	65	69	0.00042
AC068196.1	0.428571	2.4325	3.3255	40.545	0.0404805	0	0.764544	0.42331	0	1	0.337571	64	0.437571	55	20	65	67	64	68	64	64	65	6.0000e-05
XIST	0.928571	4.10382	3.2859	33.76	0.0932914	0.875	0.544126	0.418264	0.449785	0.739283	0.618863	22	0.605291	8	15	4	49	12	44	23	15	32	0.12944
RP1-239B22.5	0.5	3.02613	2.36833	38.7	0.210083	0.125	0.686254	0.301323	0.122307	0.485788	0.308056	66	0.344134	67	55	66	68	65	67	66	66	67	0
CEP83-AS1	0.571429	2.12873	2.0155	38.4038	0.122694	0.25	0.804605	0.256356	0.141946	0.65373	0.380955	63	0.421328	58	36	64	63	63	63	63	63	63	0
RP11-29G8.3	0.785714	2.48628	1.77879	37.9736	0.199148	0.625	0.75745	0.226188	0.170458	0.502481	0.468567	59	0.456315	51	48	55	61	55	61	59	56	60	0
PDXDC2P	0.928571	2.50563	1.7041	34.01	0.174302	0.875	0.754899	0.216669	0.433212	0.544094	0.597865	29	0.564775	21	22	20	43	26	46	31	27	37	0.00127
CROCCP2	1	3.00804	1.63886	33.58	0.257565	1	0.68864	0.208355	0.461717	0.422156	0.613958	25	0.556174	24	33	12	36	17	31	25	21	28	0.00352
LOC101927156	0.428571	1.06778	1.39	37.33	0.190543	0	0.944524	0.176639	0.213126	0.516273	0.318485	65	0.370113	65	30	68	64	66	66	65	65	67	0
GAS5-AS1	1	2.37351	1.37886	33.5286	0.235834	1	0.772323	0.175219	0.465126	0.449681	0.627502	20	0.57247	17	28	15	24	8	25	20	17	23	0.01169
MCM3AP-AS1	0.928571	1.57478	1.23348	35.59	0.143638	0.875	0.877661	0.156691	0.328472	0.604516	0.595516	31	0.568468	18	24	30	39	11	48	32	27	38	0.0033
CTC-487M23.5	0.785714	1.27084	1.15373	37.9482	0.141722	0.625	0.917745	0.146528	0.172146	0.60871	0.495655	54	0.494026	39	35	57	57	49	59	55	53	56	0
LINC00116	1	1.78465	0.992571	32.38	0.276375	1	0.849982	0.125989	0.541266	0.400146	0.643462	15	0.583477	14	18	14	17	7	19	15	14	16	0.01228
RP11-498C9.15	0.928571	1.37641	0.959333	33.7623	0.212322	0.875	0.903823	0.121753	0.449632	0.482478	0.605789	27	0.566537	20	19	26	29	21	38	27	25	30	0
LINC00324	0.928571	1.16813	0.88981	32.3915	0.193943	0.875	0.931291	0.112892	0.540501	0.510751	0.630512	17	0.594087	13	9	22	21	15	26	19	17	22	0.01207
HCG11	1	1.2935	0.878857	33.4557	0.213258	1	0.914756	0.111496	0.469956	0.481104	0.647352	14	0.595462	12	16	17	14	2	21	14	12	15	0.09407
SIK3-IT1	0.928571	1.43681	0.855667	35.59	0.284254	0.875	0.895857	0.108541	0.328472	0.391368	0.568211	45	0.519848	32	37	43	47	33	49	45	40	47	0
ABCA11P	1	1.30088	0.791714	35.995	0.266385	1	0.913784	0.100391	0.301624	0.411643	0.604324	28	0.545488	27	38	28	27	5	36	27	25	31	0.00017
TUG1	1	1.2962	0.780286	31.9121	0.272323	1	0.9144	0.098934	0.572281	0.404758	0.657599	11	0.598075	10	12	13	13	6	13	12	10	13	0.30025
TP73-AS1	0.928571	0.842444	0.744905	35.4369	0.188287	0.875	0.974243	0.0944249	0.33862	0.519993	0.595957	30	0.560456	22	23	39	34	13	45	31	27	37	0.00067
DLEU2	1	0.771686	0.734571	33.4621	0.152636	1	0.983574	0.0931079	0.46953	0.585543	0.667797	9	0.626351	1	10	16	11	1	16	9	6	12	0.62185
OIP5-AS1	1	0.993553	0.678857	31.0529	0.254035	1	0.954314	0.0860074	0.629244	0.426466	0.67656	5	0.619206	4	5	9	7	3	10	6	5	8	0.99998
LINC00094	1	0.982657	0.664571	31.3157	0.261293	1	0.955751	0.0841868	0.611819	0.417669	0.672118	8	0.613885	6	8	11	8	4	11	8	7	9	0.91053
MEG3	0.785714	2.84303	1.46333	35.7991	0.338486	0.625	0.710402	0.185985	0.314611	0.336841	0.463384	60	0.434568	57	50	54	60	59	60	59	58	60	0
FOXN3-AS2	0.785714	1.72001	0.9178	35.9755	0.358532	0.625	0.858508	0.11646	0.30292	0.318874	0.474965	57	0.444352	53	43	58	58	58	58	57	56	58	0
RP11-399K21.11	0.571429	2.83612	0.915	38.2125	0.5992	0.25	0.711313	0.116103	0.154624	0.158499	0.287258	67	0.278108	68	68	67	66	69	65	68	67	68	0
RP11-325K4.3	1	2.23805	0.661143	34.8771	0.555535	1	0.790188	0.0837498	0.375728	0.182126	0.568146	46	0.486358	43	57	34	45	40	43	44	42	46	0
NEAT1	1	1.77664	0.627429	29.3143	0.494473	1	0.851039	0.0794531	0.744495	0.218487	0.656846	12	0.578695	16	13	8	12	30	9	12	11	14	0.21621
AC007228.9	0.928571	1.33623	0.62019	32.5285	0.41678	0.875	0.909122	0.0785306	0.531424	0.271865	0.593502	33	0.533188	29	25	32	33	44	33	32	30	35	0
CTC-444N24.11	1	1.51862	0.536	31.6886	0.508033	1	0.885067	0.0678009	0.587102	0.210039	0.628998	18	0.550002	26	26	19	19	24	17	19	18	21	0
LRRC75A-AS1	1	2.11663	0.519714	27.2564	0.62708	1	0.8062	0.0657254	0.880913	0.144297	0.664997	10	0.579427	15	11	5	9	43	6	10	7	13	0.5589
LINC00662	1	1.62939	0.507429	35.2471	0.55392	1	0.870458	0.0641596	0.3512	0.183036	0.575467	40	0.493771	40	51	38	41	31	39	40	39	41	0
HCG18	1	0.960289	0.483143	29.7121	0.411899	1	0.958701	0.0610645	0.718121	0.275544	0.675132	6	0.602686	9	6	10	6	10	7	7	6	8	0.98605
NCBP2-AS2	1	0.888733	0.476857	28.4721	0.397145	1	0.968138	0.0602634	0.800322	0.286934	0.694438	4	0.623132	3	4	6	4	9	4	4	3	4	0.99934

gene	detectability_raw	variability_raw	effect_size_raw	mean_ct_raw	p_value_raw	detectability	inverse_variability	effect_size	inverse_mean_ct	stat_support	composite_score	rank_original	equal_score	rank_equal	rank_without_detectability	rank_without_inverse_variability	rank_without_effect_size	rank_without_inverse_mean_ct	rank_without_stat_support	MC_median_rank	MC_Q1_rank	MC_Q3_rank	MC_top10_probability
LRRC37BP1	1	1.15364	0.474571	32.495	0.473682	1	0.933201	0.0599721	0.533643	0.231901	0.628553	19	0.551743	25	27	23	18	16	18	20	18	21	0
LINC00657	1	1.20554	0.471714	28.805	0.489684	1	0.926357	0.059608	0.778257	0.221526	0.674997	7	0.59715	11	7	7	5	18	5	7	6	9	0.89862
RP11-96D1.10	1	1.61488	0.467429	35.1186	0.583176	1	0.872373	0.0590618	0.359723	0.166963	0.574928	41	0.491624	42	52	40	40	32	37	41	40	42	0
HNRNPU-AS1	1	1.19227	0.458857	33.2543	0.498457	1	0.928107	0.0579694	0.483309	0.215981	0.615475	24	0.537073	28	32	25	23	19	22	24	23	26	0
SNHG11	1	1.02304	0.454	32.2207	0.458741	1	0.950426	0.0573504	0.551825	0.241909	0.636111	16	0.560302	23	21	21	15	14	15	16	16	18	0
RP11-363E7.4	0.928571	1.67583	0.440286	35.2992	0.629716	0.875	0.864334	0.0556026	0.347747	0.142987	0.530335	50	0.457134	50	58	49	51	50	52	50	49	51	0
SENP3-EIF4A1	0.857143	1.32502	0.431	37.2875	0.587392	0.75	0.9106	0.0544192	0.215943	0.164714	0.477664	56	0.419135	59	62	59	56	56	56	57	56	58	0
CTD-3185P2.1	1	1.30705	0.418286	34.2843	0.563739	1	0.912969	0.0527988	0.415029	0.177549	0.593914	32	0.511669	34	39	33	28	27	28	31	29	33	0
LL22NC03-N27C7.1	1	1.18775	0.366	34.6479	0.589213	1	0.928703	0.0461352	0.390928	0.163747	0.589528	36	0.505903	35	42	36	30	25	30	34	32	35	0
ZFAS1	1	0.970018	0.286857	26.2686	0.630453	1	0.957418	0.0360488	0.946399	0.142622	0.702235	2	0.616498	5	2	3	2	23	3	3	2	3	0.98443
MZF1-AS1	1	0.778894	0.266	35.4879	0.615714	1	0.982624	0.0333907	0.335243	0.150009	0.585252	37	0.500253	37	44	44	32	20	32	36	33	38	0
HOTAIR	0.785714	1.88795	0.477133	37.45	0.66134	0.625	0.83636	0.0602986	0.205171	0.127686	0.420634	62	0.370903	64	65	63	62	62	62	62	62	62	0
SNHG20	1	1.08926	0.292571	35.9929	0.647951	1	0.941692	0.0367771	0.301766	0.134073	0.569454	44	0.482862	45	56	46	42	29	40	44	42	45	0
PSMA3-AS1	1	1.71292	0.256	32.4686	0.775187	1	0.859443	0.0321162	0.535395	0.0780862	0.593199	35	0.501008	36	41	29	26	46	24	32	28	35	0
LOC653160	1	1.25916	0.226857	34.85	0.748626	1	0.919285	0.0284021	0.377527	0.0889735	0.57394	42	0.482838	46	53	42	37	36	35	42	39	43	0
GAS5	1	0.768138	0.216	25.5557	0.681967	1	0.984042	0.0270184	0.993655	0.118095	0.712753	1	0.624562	2	1	1	1	22	1	1	1	1	0.99087
JPX	1	1.2383	0.206	33.9964	0.768481	1	0.922036	0.025744	0.434111	0.0807994	0.584458	38	0.492538	41	45	41	31	37	27	37	34	38	0
EPB41L4A-AS1	1	0.829754	0.204	29.5686	0.709604	1	0.975916	0.0254891	0.727639	0.10569	0.656378	13	0.566947	19	14	18	10	28	8	12	10	14	0.32607
FGD5-AS1	0.928571	0.777385	0.195667	30.3254	0.726289	0.875	0.982823	0.024427	0.677469	0.0984324	0.609287	26	0.53163	30	17	31	22	48	20	26	23	31	0
SNHG7	1	1.54472	0.174	33.5179	0.833069	1	0.881626	0.0216657	0.465836	0.0555987	0.579385	39	0.484945	44	49	37	35	45	29	39	36	42	0
SNHG5	1	1.10124	0.166857	25.46	0.796393	1	0.940112	0.0207554	1	0.0696585	0.699139	3	0.606105	7	3	2	3	35	2	3	2	4	0.93793
RP11-282O18.3	0.928571	1.50687	0.161952	37.1708	0.847681	0.875	0.886617	0.0201303	0.223681	0.050169	0.493603	55	0.41112	62	66	56	54	52	54	55	54	56	0
SDCBP2-AS1	1	1.26906	0.146	34.6393	0.837647	1	0.91798	0.0180972	0.391496	0.0538874	0.570903	43	0.476292	47	54	45	38	41	34	44	41	45	0
RP11-84C13.1	1	0.944904	0.131143	36.3121	0.823755	1	0.96073	0.0162038	0.2806	0.0591098	0.557418	47	0.463329	49	61	48	44	34	41	47	46	47	0
NUTM2A-AS1	1	1.21769	0.129714	32.9886	0.851354	1	0.924755	0.0160217	0.500923	0.0488189	0.593222	34	0.498104	38	40	35	25	39	23	32	28	35	0
RP11-473M20.16	0.785714	0.647138	0.110667	34.8009	0.850124	0.625	1	0.0135942	0.380782	0.0492703	0.471302	58	0.413729	61	47	62	55	60	55	58	56	60	0
RP11-363G2.4	0.571429	1.47148	0.1545	38.8525	0.883936	0.25	0.891284	0.0191805	0.112198	0.0370911	0.283242	69	0.261951	69	69	69	65	68	64	68	67	69	0
MALAT1	0.928571	1.46554	0.132571	34.7177	0.872449	0.875	0.892068	0.0163858	0.386298	0.0411756	0.525568	51	0.442185	54	59	50	50	53	50	51	50	51	0
ZNRD1-AS1	0.928571	1.29772	0.0981429	35.3169	0.89669	0.875	0.9142	0.0119981	0.346575	0.0326176	0.520316	52	0.436078	56	60	52	52	51	51	52	52	52	0
SNHG16	1	0.99564	0.066	30.7507	0.913505	1	0.954039	0.00790161	0.649273	0.0268161	0.624924	21	0.527606	31	29	24	16	38	12	20	17	24	1.0000e-05
RP11-473I1.10	0.857143	0.95514	0.0326667	31.5467	0.960023	0.75	0.95938	0.00365343	0.596509	0.0113058	0.538039	49	0.46417	48	34	51	48	57	47	49	47	51	0
RP11-819C2	0.857143	2.0413	0.021	36.2383	0.984985	0.75	0.816135	0.0021665	0.285493	0.0032902	0.446088	61	0.371417	63	67	61	59	61	57	61	60	61	0

gene	detectability_raw	variability_raw	effect_size_raw	mean_ct_raw	p_value_raw	detectability	inverse_variability	effect_size	inverse_mean_ct	stat_support	composite_score	rank_original	equal_score	rank_equal	rank_without_detectability	rank_without_inverse_variability	rank_without_effect_size	rank_without_inverse_mean_ct	rank_without_stat_support	MC_median_rank	MC_Q1_rank	MC_Q3_rank	MC_top10_probability
1.1								7		5													
RP11-38P22.2	0.928571	1.42447	0.0164762	36.0446	0.983746	0.875	0.897484	0.00159003	0.298335	0.00368324	0.50235	53	0.415218	60	64	53	53	54	53	53	53	54	0
LINC00667	1	0.970069	0.00971429	31.2229	0.987047	1	0.957411	0.000728259	0.617974	0.00263701	0.615486	23	0.51575	33	31	27	20	42	14	24	21	29	0
AC000120.7	1	1.22768	0.004	35.5714	0.995418	1	0.923437	0	0.329703	0	0.550628	48	0.450628	52	63	47	46	47	42	48	48	49	0

Notes. The prioritization score was used for candidate selection only and not as confirmatory differential-expression evidence. MC, Monte Carlo; Q1, first quartile; Q3, third quartile. Rank 1 denotes the highest-priority candidate.

Supplementary Table S1. Robustness of BRD3OS (LINC00094) candidate prioritization across alternative weighting schemes.

original_rank	equal_weight_rank	MC_median_rank	MC_Q1_rank	MC_Q3_rank	MC_top10_probability	rank_without_detectability	rank_without_inverse_variability	rank_without_effect_size	rank_without_inverse_mean_ct	rank_without_stat_support
8	6	8	7	9	0.91053	8	11	8	4	11

Notes. BRD3OS ranks under the original five-component weighting scheme, equal weighting, leave-one-component-out analyses, and 100,000 Monte Carlo perturbations are shown. MC, Monte Carlo; Q1, first quartile; Q3, third quartile.

Supplementary Data S2. Fragment-overlap consistency analysis of BRD3OS DRACH candidate positions.

a_pos_full	motif_seq	fragment_A	fragment_B	paired_status_A	paired_status_B	paired_status_concordant	local_context_A	local_context_B	local_context_concordant	exact_u1_A	exact_u1_B	exact_u5_A	exact_u5_B	exact_u10_A	exact_u10_B	mean_u1_pm10_A	mean_u1_pm10_B	mean_u5_pm10_A	mean_u5_pm10_B	mean_u10_pm10_A	mean_u10_pm10_B	final_composite_A	final_composite_B
2671	AGACA	fragment01	fragment02	paired	paired	True	stem	stem	True	0.178846	0.178846	0.00318291	0.00318291	0.00175739	0.00175739	0.459346	0.459346	0.0627575	0.0627575	0.0148825	0.0148825	0.139489	0.139489
2706	GAACU	fragment01	fragment02	unpaired	unpaired	True	hairpin_loop	internal_or_bulge	False	0.942743	0.942743	0.428753	0.428753	0.0151445	0.0151445	0.570868	0.570868	0.17181	0.17181	0.0358165	0.0358165	0.543265	0.543265
2799	UAACC	fragment01	fragment02	unpaired	unpaired	True	internal_or_bulge	internal_or_bulge	True	0.68492	0.68492	0.33579	0.33579	0.0482823	0.0482823	0.433092	0.433092	0.158545	0.158545	0.0594869	0.0594869	0.41229	0.41229
2806	UGACU	fragment01	fragment02	unpaired	paired	False	internal_or_bulge	stem	False	0.88086	0.88086	0.231837	0.231837	0.130721	0.130721	0.540513	0.540513	0.196652	0.196652	0.0706953	0.0706953	0.483609	0.483609
2845	UGACA	fragment01	fragment02	paired	paired	True	stem	stem	True	0.0442297	0.0442297	0.00341225	0.00341225	2.0635e-05	2.0635e-05	0.324495	0.322977	0.0950743	0.0950535	0.00101125	0.00101125	0.0745677	0.074338
2872	AAACA	fragment01	fragment02	unpaired	paired	False	internal_or_bulge	stem	False	0.624134	0.61315	0.0216431	0.03485	0.00409255	0.0041829	0.337748	0.350019	0.0406201	0.0421081	0.00600787	0.00577855	0.279292	0.280736
2957	GAACU	fragment01	fragment02	unpaired	unpaired	True	internal_or_bulge	internal_or_bulge	True	0.198257	0.383192	0.0985574	0.0629794	0.00601218	0.00249285	0.391551	0.352042	0.0961067	0.0626001	0.0335984	0.0212961	0.164654	0.210242
5039	GGACA	fragment02	fragment03	paired	paired	True	stem	stem	True	0.267689	0.350389	0.00127289	0.00444414	1.5353e-05	1.3795e-05	0.490694	0.518705	0.110266	0.125998	0.00811864	0.0192816	0.179048	0.215118
5089	UGACU	fragment02	fragment03	paired	paired	True	stem	stem	True	0.336414	0.247356	0.062211	0.0471702	0.00939425	0.00854009	0.454345	0.410434	0.110421	0.0674167	0.0267374	0.0105179	0.214768	0.168054
5144	AAACU	fragment02	fragment03	unpaired	unpaired	True	internal_or_bulge	internal_or_bulge	True	0.578047	0.560481	0.19521	0.186902	0.0123327	0.0127739	0.550252	0.54752	0.158502	0.160577	0.0363857	0.0389593	0.352559	0.344305
5157	AAACC	fragment02	fragment03	unpaired	unpaired	True	internal_or_bulge	internal_or_bulge	True	0.986947	0.986947	0.648119	0.648119	0.0563615	0.0563615	0.550919	0.550241	0.202675	0.201869	0.0228567	0.0225544	0.617146	0.616948
5175	GGACA	fragment02	fragment03	unpaired	paired	False	internal_or_bulge	stem	False	0.546967	0.546967	0.0491091	0.0491091	0.00348227	0.00348227	0.496262	0.496262	0.168544	0.168544	0.0246244	0.0246244	0.296589	0.296589
5196	UGACA	fragment02	fragment03	paired	paired	True	stem	stem	True	0.118079	0.118079	0.0437139	0.0437139	0.0445687	0.0445687	0.552868	0.552868	0.25717	0.25717	0.0922472	0.0922472	0.169973	0.169973
5302	GAACC	fragment02	fragment03	unpaired	unpaired	True	internal_or_bulge	internal_or_bulge	True	0.962175	0.962175	0.516747	0.516747	0.142962	0.142962	0.682233	0.682233	0.274476	0.274476	0.0957096	0.0957096	0.614812	0.614812

Notes. DRACH positions independently represented in adjacent overlapping transcript fragments were compared for discrete RNAfold-derived structural classification and quantitative RNAplfold-derived accessibility estimates. Concordance variables indicate whether paired/unpaired status and local structural context were reproduced across fragment representations. The same full-transcript coordinate may intentionally occur in more than one fragment.

Supplementary Data S3. Complete site-level structural accessibility and composite-score sensitivity analysis of BRD3OS DRACH candidates.

site_id	fragment_id	motif_seq	a_pos_fragment	a_pos_full	paired_status	local_context	exact_u1	exact_u5	exact_u10	mean_u1_pm10	mean_u5_pm10	mean_u10_pm10	final_composite_raw	final_composite_normalized	final_rank	final_top_decile	equal_weight_score	equal_weight_rank	equal_weight_top_decile	LOO_without_exact_u1_rank	LOO_without_exact_u1_top_decile	LOO_without_exact_u5_rank	LOO_without_exact_u5_top_decile	LOO_without_exact_u10_rank	LOO_without_exact_u10_top_decile	LOO_without_mean_u1_pm10_rank	LOO_without_mean_u1_pm10_top_decile	LOO_without_mean_u5_pm10_rank	LOO_without_mean_u5_pm10_top_decile	LOO_without_mean_u10_pm10_rank	LOO_without_mean_u10_pm10_top_decile	MC_median_rank	MC_Q1_rank	MC_Q3_rank	MC_top_decile_probability
fragment01_A1117	fragment01	GAACC	1117	1117	unpaired	internal_or_bulge	0.99874	0.0032647	0.000161799	0.401086	0.0130364	0.00263885	0.411199	0.527443	48	False	0.236488	64	False	107	False	33	False	46	False	44	False	41	False	46	False	46	43	51	0
fragment01_A237	fragment01	UGACU	237	237	unpaired	internal_or_bulge	0.996241	0.013592	0.00874105	0.572669	0.164646	0.0110173	0.455872	0.595242	36	False	0.294484	39	False	64	False	17	False	35	False	38	False	35	False	35	False	35	33	37	0
fragment01_A1912	fragment01	AAACC	1912	1912	unpaired	hairpin_loop	0.994454	0.0226954	0.0113769	0.443352	0.0641776	0.0386001	0.429721	0.554838	40	False	0.262443	50	False	88	False	29	False	40	False	39	False	39	False	39	False	39	38	42	0
fragment03_A5620	fragment03	AAACU	620	5620	unpaired	hairpin_loop	0.99141	0.0245666	0.0232341	0.609187	0.374743	0.0852592	0.488574	0.645767	28	False	0.3514	27	False	43	False	9	True	25	False	30	False	32	False	27	False	28	25	32	5.0000e-05
fragment02_A3143	fragment02	GGACC	643	3143	unpaired	hairpin_loop	0.98911	0.0303359	0.00137778	0.331167	0.00805688	0.00103119	0.404443	0.515783	50	False	0.226846	67	False	115	False	41	False	50	False	41	False	45	False	50	False	50	47	53	0
fragment02_A5157	fragment02	AAACC	2657	5157	unpaired	internal_or_bulge	0.986947	0.648119	0.0563615	0.550919	0.202675	0.0228567	0.617146	0.844412	8	True	0.411313	17	False	17	False	13	False	7	True	7	True	7	True	7	True	8	7	10	0.95751
fragment0	fragment	AAACC	157	5157	unpaired	internal	0.986947	0.648119	0.056361	0.550241	0.201869	0.022554	0.616948	0.844107	9	True	0.411015	18	False	18	False	14	False	8	True	8	True	8	True	8	True	9	8	11	0.91228

site_id	fragment_id	motif_seq	a_pos_fragment	a_pos_full	paired_status	local_context	exact_u1	exact_u5	exact_u10	mean_u1_pm10	mean_u5_pm10	mean_u10_pm10	final_composite_raw	final_composite_normalized	final_rank	final_top_decile	equal_weight_score	equal_weight_rank	equal_weight_top_decile	LOO_without_exact_u1_rank	LOO_without_exact_u1_top_decile	LOO_without_exact_u5_rank	LOO_without_exact_u5_top_decile	LOO_without_exact_u10_rank	LOO_without_exact_u10_top_decile	LOO_without_mean_u1_pm10_rank	LOO_without_mean_u1_pm10_top_decile	LOO_without_mean_u5_pm10_rank	LOO_without_mean_u5_pm10_top_decile	LOO_without_mean_u10_pm10_rank	LOO_without_mean_u10_pm10_top_decile	MC_median_rank	MC_Q1_rank	MC_Q3_rank	MC_top_decile_probability
3_A5157	03					_or_bulge			5			4																							
fragment03_A5673	fragment03	UAACA	673	5673	unpaired	internal_or_bulge	0.986839	0.0258637	0.0106036	0.706279	0.442931	0.138322	0.510071	0.67898	25	False	0.38514	22	False	37	False	3	True	23	False	25	False	26	False	24	False	25	23	27	0.00544
fragment01_A1423	fragment01	UAACC	1423	1423	unpaired	internal_or_bulge	0.98377	0.122892	0.0145091	0.58443	0.172446	0.0529305	0.484049	0.638776	29	False	0.32183	33	False	44	False	15	False	26	False	31	False	25	False	28	False	29	26	31	0
fragment01_A301	fragment01	GGACC	301	301	unpaired	internal_or_bulge	0.966759	0.00194126	0.000661298	0.468318	0.0921444	0.0052231	0.41864	0.537719	42	False	0.25841	54	False	90	False	30	False	42	False	45	False	42	False	42	False	43	41	48	0
fragment01_A1042	fragment01	AAACA	1042	1042	unpaired	hairpin_loop	0.966508	0.943544	0.119462	0.6118	0.337626	0.124121	0.717849	1	1	True	0.517177	6	True	4	True	10	True	2	True	1	True	1	True	1	True	1	1	2	1
fragment02_A3847	fragment02	AGACU	1347	3847	unpaired	hairpin_loop	0.965737	0.553759	0.000883833	0.33882	0.0708239	0.00822668	0.534768	0.717137	22	False	0.322944	32	False	28	False	43	False	19	False	13	False	17	False	22	False	22	19	25	0.00723
fragment02_A5302	fragment02	GAACC	2802	5302	unpaired	internal_or_bulge	0.962175	0.516747	0.142962	0.682233	0.274476	0.0957096	0.614812	0.840807	10	True	0.445717	13	False	15	False	6	True	9	True	10	True	9	True	9	True	9	8	10	0.99811
frag	fra	GAA	302	530	unp	int	0.9	0.5	0.1	0.6	0.2	0.0	0.61	0.8408	11	Tru	0.44	14	False	16	False	7	True	10	True	11	True	10	True	10	True	10	9	11	0.9896

site_id	fragment_id	motif_seq	a_pos_fragment	a_pos_full	paired_status	local_context	exact_u1	exact_u5	exact_u10	mean_u1_pm10	mean_u5_pm10	mean_u10_pm10	final_composite_raw	final_composite_normalized	final_rank	final_top_decile	equal_weight_score	equal_weight_rank	equal_weight_top_decile	LOO_without_exact_u1_rank	LOO_without_exact_u1_top_decile	LOO_without_exact_u5_rank	LOO_without_exact_u5_top_decile	LOO_without_exact_u10_rank	LOO_without_exact_u10_top_decile	LOO_without_mean_u1_pm10_rank	LOO_without_mean_u1_pm10_top_decile	LOO_without_mean_u5_pm10_rank	LOO_without_mean_u5_pm10_top_decile	LOO_without_mean_u10_pm10_rank	LOO_without_mean_u10_pm10_top_decile	MC_median_rank	MC_Q1_rank	MC_Q3_rank	MC_top_decile_probability
ment03_A5302	gment03	CC		2	aired	ernal_or_bulge	62175	16747	42962	82233	74476	957096	4812	07		e	5717																		4
fragment01_A368	fragment01	GAACA	368	368	unpaired	internal_or_bulge	0.953145	0.162653	0.000492647	0.452638	0.0944808	0.00857617	0.452086	0.589392	37	False	0.278664	43	False	54	False	32	False	36	False	33	False	33	False	37	False	36	35	37	0
fragment02_A3767	fragment02	AAACC	1267	3767	unpaired	internal_or_bulge	0.953028	0.527604	0.0244556	0.561517	0.251154	0.0747728	0.580986	0.788544	12	True	0.398748	21	False	22	False	18	False	11	True	12	True	12	True	12	True	13	12	15	0.47435
fragment03_A5677	fragment03	AAACU	677	5677	paired	stem	0.946141	0.908712	0.0174581	0.698229	0.418537	0.133917	0.713357	0.993061	2	True	0.520499	5	True	3	True	5	True	1	True	2	True	2	True	2	True	2	2	2	1
fragment03_A5604	fragment03	UAACU	604	5604	unpaired	internal_or_bulge	0.944878	3.1065e-05	5.1229e-06	0.465966	0.164396	0.0191109	0.418006	0.536738	43	False	0.265731	48	False	83	False	31	False	43	False	46	False	46	False	43	False	45	42	48	0
fragment01_A2706	fragment01	GAACU	2706	2706	unpaired	hairpin_loop	0.942743	0.428753	0.0151445	0.570868	0.17181	0.0358165	0.543265	0.730265	20	False	0.360856	25	False	25	False	23	False	17	False	20	False	18	False	20	False	20	19	21	0.00142
fragment02_A2706	fragment02	GAACU	206	2706	unpaired	internal_or_bul	0.942743	0.428753	0.0151445	0.570868	0.17181	0.0358165	0.543265	0.730265	21	False	0.360856	26	False	26	False	24	False	18	False	21	False	19	False	21	False	21	20	22	0.00044

site_id	fragment_id	motif_seq	a_pos_fragment	a_pos_full	paired_status	local_context	exact_u1	exact_u5	exact_u10	mean_u1_pm10	mean_u5_pm10	mean_u10_pm10	final_composite_raw	final_composite_normalized	final_rank	final_top_decile	equal_weight_score	equal_weight_rank	equal_weight_top_decile	LOO_without_exact_u1_rank	LOO_without_exact_u1_top_decile	LOO_without_exact_u5_rank	LOO_without_exact_u5_top_decile	LOO_without_exact_u10_rank	LOO_without_exact_u10_top_decile	LOO_without_mean_u1_pm10_rank	LOO_without_mean_u1_pm10_top_decile	LOO_without_mean_u5_pm10_rank	LOO_without_mean_u5_pm10_top_decile	LOO_without_mean_u10_pm10_rank	LOO_without_mean_u10_pm10_top_decile	MC_median_rank	MC_Q1_rank	MC_Q3_rank	MC_top_decile_probability
						ge																													
fragment01_A2342	fragment01	GAACC	2342	2342	unpaired	internal_or_bulge	0.9424478	0.1068619	5.69775e-06	0.3743392	0.0422407	0.000766593	0.416994	0.535175	44	False	0.24445	60	False	84	False	45	False	44	False	40	False	40	False	44	False	45	42	47	0
fragment02_A3672	fragment02	GAACC	1172	3672	unpaired	hairpin_loop	0.937308	0.0529743	0.000225203	0.589564	0.28449	0.0814687	0.462281	0.605144	33	False	0.324339	31	False	48	False	19	False	32	False	37	False	37	False	33	False	33	32	35	0
fragment02_A3160	fragment02	UGACA	660	3160	unpaired	internal_or_bulge	0.932966	0.00249165	5.7148e-05	0.421896	0.0673216	0.00416509	0.397391	0.504889	51	False	0.23815	62	False	100	False	42	False	51	False	51	False	51	False	51	False	51	49	55	0
fragment01_A1654	fragment01	AAACU	1654	1654	unpaired	internal_or_bulge	0.918025	0.341035	0.21829	0.536237	0.18114	0.0628901	0.53009	0.709911	23	False	0.376269	24	False	27	False	20	False	24	False	22	False	23	False	23	False	23	22	24	0
fragment02_A3884	fragment02	UGACU	1384	3884	unpaired	hairpin_loop	0.916607	0.00926308	0.000787987	0.528193	0.248511	0.0813711	0.431356	0.557364	39	False	0.297456	38	False	59	False	26	False	39	False	43	False	43	False	40	False	39	38	42	0
fragment01_A600	fragment01	UGACC	600	600	unpaired	internal_or_bulge	0.91093	0.0877684	0.000189921	0.43434	0.129527	0.00879653	0.41933	0.538784	41	False	0.261925	51	False	75	False	40	False	41	False	42	False	44	False	41	False	44	43	45	0
fragment01_	fragment01	UGACA	1341	1341	paired	stem	0.903978	0.0408194	0.000402018	0.417451	0.0590973	0.0154336	0.395936	0.50264	52	False	0.23953	61	False	93	False	46	False	52	False	52	False	52	False	52	False	52	50	55	0

site_id	fragment_id	motif_seq	a_pos_fragment	a_pos_full	paired_status	local_context	exact_u1	exact_u5	exact_u10	mean_u1_pm10	mean_u5_pm10	mean_u10_pm10	final_composite_raw	final_composite_normalized	final_rank	final_top_decile	equal_weight_score	equal_weight_rank	equal_weight_top_decile	LOO_without_exact_u1_rank	LOO_without_exact_u1_top_decile	LOO_without_exact_u5_rank	LOO_without_exact_u5_top_decile	LOO_without_exact_u10_rank	LOO_without_exact_u10_top_decile	LOO_without_mean_u1_pm10_rank	LOO_without_mean_u1_pm10_top_decile	LOO_without_mean_u5_pm10_rank	LOO_without_mean_u5_pm10_top_decile	LOO_without_mean_u10_pm10_rank	LOO_without_mean_u10_pm10_top_decile	MC_median_rank	MC_Q1_rank	MC_Q3_rank	MC_top_decile_probability
A1341									7																										
fragment02_A4548	fragment02	AAACU	2048	4548	unpaired	internal_or_bulge	0.888489	0.337201	0.164092	0.686725	0.409324	0.177886	0.564516	0.763099	16	False	0.443953	15	False	21	False	11	True	16	False	19	False	20	False	16	False	16	14	18	0.04474
fragment01_A2806	fragment01	UGACU	2806	2806	unpaired	internal_or_bulge	0.888086	0.231837	0.130721	0.540513	0.196652	0.0706953	0.483609	0.638096	30	False	0.341888	29	False	32	False	27	False	30	False	26	False	27	False	30	False	29	28	29	0
fragment02_A2806	fragment02	UGACU	306	2806	paired	stem	0.888086	0.231837	0.130721	0.540513	0.196652	0.0706953	0.483609	0.638096	31	False	0.341888	30	False	33	False	28	False	31	False	27	False	28	False	31	False	30	29	30	0
fragment02_A3945	fragment02	GAACU	1445	3945	unpaired	internal_or_bulge	0.875706	0.491548	0.120856	0.625592	0.238388	0.0537302	0.561834	0.758954	17	False	0.400097	20	False	20	False	21	False	15	False	17	False	14	False	15	False	17	15	18	0.00021
fragment02_A4807	fragment02	GAACA	2307	4807	unpaired	internal_or_bulge	0.867723	0.680208	0.080815	0.773748	0.497728	0.269978	0.66117	0.912431	5	True	0.528367	4	True	6	True	4	True	3	True	4	True	4	True	4	True	5	4	5	1
fragment01_A1263	fragment01	GAACC	1263	1263	unpaired	internal_or_bulge	0.862214	0.300208	0.0385151	0.5173	0.167738	0.0424514	0.47717	0.628147	32	False	0.321404	34	False	31	False	38	False	29	False	29	False	29	False	32	False	31	30	32	0
fragme	fragm	AGACA	1825	4325	unpair	inter	0.861	0.266	3.596	0.382	0.094	0.025	0.4363	0.565005	38	False	0.2717	46	False	47	False	50	False	38	False	35	False	38	False	38	False	39	38	41	0

site_id	fragment_id	motif_seq	a_pos_fragment	a_pos_full	paired_status	local_context	exact_u1	exact_u5	exact_u10	mean_u1_pm10	mean_u5_pm10	mean_u10_pm10	final_composite_raw	final_composite_normalized	final_rank	final_top_decile	equal_weight_score	equal_weight_rank	equal_weight_top_decile	LOO_without_exact_u1_rank	LOO_without_exact_u1_top_decile	LOO_without_exact_u5_rank	LOO_without_exact_u5_top_decile	LOO_without_exact_u10_rank	LOO_without_exact_u10_top_decile	LOO_without_mean_u1_pm10_rank	LOO_without_mean_u1_pm10_top_decile	LOO_without_mean_u5_pm10_rank	LOO_without_mean_u5_pm10_top_decile	LOO_without_mean_u10_pm10_rank	LOO_without_mean_u10_pm10_top_decile	MC_median_rank	MC_Q1_rank	MC_Q3_rank	MC_top_decile_probability
nt02_A4325	ent02				ed	nal_or_bulge	717	483	2e-05	6	03004	6532	01				53																		
fragment02_A4576	fragment02	UAACU	2076	4576	unpaired	internal_or_bulge	0.857727	0.0480665	0.0385128	0.539756	0.182031	0.0262654	0.416552	0.534492	45	False	0.282806	42	False	56	False	35	False	45	False	50	False	48	False	45	False	46	43	47	0
fragment01_A1221	fragment01	AAACU	1221	1221	paired	stem	0.853406	0.101379	0.0440661	0.464506	0.0970772	0.0547186	0.410563	0.525239	49	False	0.269192	47	False	58	False	47	False	49	False	49	False	47	False	49	False	48	47	48	0
fragment02_A4115	fragment02	AAACU	1615	4115	unpaired	internal_or_bulge	0.845875	0.590038	0.0194681	0.529433	0.253787	0.0658356	0.553598	0.74623	19	False	0.384073	23	False	19	False	34	False	14	False	15	False	16	False	18	False	19	17	20	0
fragment02_A4721	fragment02	GAACA	2221	4721	unpaired	internal_or_bulge	0.843107	0.218499	0.0772971	0.540155	0.194835	0.0187453	0.458886	0.599898	34	False	0.31544	35	False	38	False	36	False	37	False	36	False	36	False	34	False	35	34	35	0
fragment02_A4479	fragment02	AAACA	1979	4479	unpaired	internal_or_bulge	0.841439	0.722954	0.19435	0.662039	0.340091	0.119128	0.633949	0.870373	6	True	0.48	10	True	8	True	16	False	6	True	6	True	5	True	6	True	7	6	7	0.99971
fragment03_A5651	fragment03	AAACA	651	5651	unpaired	internal_or_bul	0.810443	0.632746	0.516898	0.739552	0.554579	0.344797	0.677162	0.937138	3	True	0.599836	2	True	2	True	2	True	4	True	3	True	3	True	3	True	3	3	4	1

site_id	fragment_id	motif_seq	a_pos_fragment	a_pos_full	paired_status	local_context	exact_u1	exact_u5	exact_u10	mean_u1_pm10	mean_u5_pm10	mean_u10_pm10	final_composite_raw	final_composite_normalized	final_rank	final_top_decile	equal_weight_score	equal_weight_rank	equal_weight_top_decile	LOO_without_exact_u1_rank	LOO_without_exact_u1_top_decile	LOO_without_exact_u5_rank	LOO_without_exact_u5_top_decile	LOO_without_exact_u10_rank	LOO_without_exact_u10_top_decile	LOO_without_mean_u1_pm10_rank	LOO_without_mean_u1_pm10_top_decile	LOO_without_mean_u5_pm10_rank	LOO_without_mean_u5_pm10_top_decile	LOO_without_mean_u10_pm10_rank	LOO_without_mean_u10_pm10_top_decile	MC_median_rank	MC_Q1_rank	MC_Q3_rank	MC_top_decile_probability
						ge																													
fragment03_A5514	fragment03	AGACC	514	5514	unpaired	internal_or_bulge	0.798768	0.0536664	0.00956777	0.517251	0.152291	0.048265	0.389172	0.492219	54	False	0.263301	49	False	60	False	49	False	54	False	54	False	53	False	53	False	53	52	55	0
fragment02_A4539	fragment02	AAACA	2039	4539	unpaired	internal_or_bulge	0.790239	0.674786	0.0607828	0.596704	0.240363	0.067254	0.568263	0.768888	14	False	0.405021	19	False	14	False	39	False	13	False	14	False	13	False	13	False	15	13	17	0.10186
fragment02_A3816	fragment02	AGACU	1316	3816	unpaired	internal_or_bulge	0.767473	0.135634	0.0293984	0.436783	0.107586	0.0590956	0.384695	0.485272	56	False	0.255995	53	False	57	False	52	False	55	False	53	False	54	False	55	False	54	54	56	0
fragment01_A396	fragment01	GGACA	396	396	unpaired	internal_or_bulge	0.76588	0.00977481	0.00016403	0.316318	0.0477336	0.00329117	0.322904	0.389804	68	False	0.190527	81	False	118	False	61	False	68	False	63	False	67	False	69	False	69	67	71	0
fragment01_A148	fragment01	GAACC	148	148	unpaired	internal_or_bulge	0.758428	0.00307257	3.0102e-05	0.347263	0.0449435	0.000363565	0.322823	0.389679	69	False	0.19235	80	False	116	False	60	False	69	False	64	False	66	False	68	False	69	67	70	0
fragment02_A3324	fragment02	AGACU	824	3324	unpaired	internal_or_bulge	0.751525	0.0946203	0.000156085	0.40596	0.0453349	0.0118496	0.352725	0.435878	62	False	0.218241	70	False	80	False	56	False	61	False	61	False	61	False	62	False	63	60	65	0
fragmen	fragme	GAACC	1437	3937	unpaire	intern	0.7506	0.4102	0.0059	0.4931	0.1532	0.0315	0.45678	0.596648	35	False	0.30749	36	False	29	False	51	False	34	False	34	False	34	False	36	False	35	33	37	0

site_id	fragment_id	motif_seq	a_pos_fragment	a_pos_full	paired_status	local_context	exact_u1	exact_u5	exact_u10	mean_u1_pm10	mean_u5_pm10	mean_u10_pm10	final_composite_raw	final_composite_normalized	final_rank	final_top_decile	equal_weight_score	equal_weight_rank	equal_weight_top_decile	LOO_without_exact_u1_rank	LOO_without_exact_u1_top_decile	LOO_without_exact_u5_rank	LOO_without_exact_u5_top_decile	LOO_without_exact_u10_rank	LOO_without_exact_u10_top_decile	LOO_without_mean_u1_pm10_rank	LOO_without_mean_u1_pm10_top_decile	LOO_without_mean_u5_pm10_rank	LOO_without_mean_u5_pm10_top_decile	LOO_without_mean_u10_pm10_rank	LOO_without_mean_u10_pm10_top_decile	MC_median_rank	MC_Q1_rank	MC_Q3_rank	MC_top_decile_probability
t02_A3937	nt02				d	al_or_bulge	78	64	7989	72	53	936	2																						
fragment01_A2179	fragment01	AAACA	2179	2179	unpaired	internal_or_bulge	0.740502	0.540197	0.484106	0.858805	0.700207	0.599465	0.669337	0.925048	4	True	0.647088	1	True	1	True	1	True	5	True	5	True	6	True	5	True	4	3	5	0.999941
fragment02_A4580	fragment02	UGACA	2080	4580	unpaired	internal_or_bulge	0.7344	0.119591	0.00643403	0.499767	0.162204	0.0314725	0.38034	0.478544	57	False	0.258978	52	False	52	False	53	False	56	False	55	False	56	False	56	False	55	55	57	0
fragment02_A4819	fragment02	AAACA	2319	4819	unpaired	internal_or_bulge	0.730611	0.583342	0.525378	0.664176	0.539247	0.43705	0.62949	0.863485	7	True	0.579967	3	True	5	True	8	True	12	True	9	True	11	True	11	True	7	6	11	0.98348
fragment02_A3271	fragment02	AAACU	771	3271	unpaired	internal_or_bulge	0.72214	0.115442	0.0487636	0.43684	0.0620795	0.0138289	0.358911	0.445436	60	False	0.233182	66	False	66	False	55	False	60	False	58	False	58	False	60	False	60	58	62	0
fragment02_A4437	fragment02	AAACA	1937	4437	unpaired	internal_or_bulge	0.717494	0.468563	0.449209	0.713644	0.436863	0.245654	0.5762	0.781151	13	False	0.505238	8	True	9	True	12	True	20	False	16	False	15	False	14	False	14	12	16	0.33978
fragment01_A332	fragment01	GAACA	332	332	unpaired	internal_or_bu	0.703491	0.176149	0.010194	0.407204	0.0908264	0.0253109	0.362707	0.451301	59	False	0.235529	65	False	55	False	62	False	59	False	56	False	57	False	59	False	59	58	60	0

site_id	fragment_id	motif_seq	a_pos_fragment	a_pos_full	paired_status	local_context	exact_u1	exact_u5	exact_u10	mean_u1_pm10	mean_u5_pm10	mean_u10_pm10	final_composite_raw	final_composite_normalized	final_rank	final_top_decile	equal_weight_score	equal_weight_rank	equal_weight_top_decile	LOO_without_exact_u1_rank	LOO_without_exact_u1_top_decile	LOO_without_exact_u5_rank	LOO_without_exact_u5_top_decile	LOO_without_exact_u10_rank	LOO_without_exact_u10_top_decile	LOO_without_mean_u1_pm10_rank	LOO_without_mean_u1_pm10_top_decile	LOO_without_mean_u5_pm10_rank	LOO_without_mean_u5_pm10_top_decile	LOO_without_mean_u10_pm10_rank	LOO_without_mean_u10_pm10_top_decile	MC_median_rank	MC_Q1_rank	MC_Q3_rank	MC_top_decile_probability
						lge																													
fragment01_A2799	fragment01	UAACC	2799	2799	unpaired	internal_or_bulge	0.68492	0.33579	0.0482823	0.433092	0.158545	0.0594869	0.41229	0.527907	46	False	0.286686	40	False	34	False	57	False	47	False	47	False	49	False	47	False	47	40	51	0
fragment02_A2799	fragment02	UAACC	299	2799	unpaired	internal_or_bulge	0.68492	0.33579	0.0482823	0.433092	0.158545	0.0594869	0.41229	0.527907	47	False	0.286686	41	False	35	False	58	False	48	False	48	False	50	False	48	False	48	41	52	0
fragment01_A1287	fragment01	UGACU	1287	1287	paired	stem	0.683868	0.00611125	0.000939664	0.413411	0.0544406	0.000886474	0.308476	0.367512	72	False	0.193276	79	False	101	False	64	False	72	False	72	False	71	False	72	False	72	71	74	0
fragment01_A1996	fragment01	AGACU	1996	1996	unpaired	internal_or_bulge	0.673205	0.478998	0.397661	0.732038	0.403941	0.196769	0.555176	0.748667	18	False	0.480435	9	True	10	True	25	False	22	False	23	False	22	False	19	False	18	16	22	0.03538
fragment02_A4442	fragment02	AAACA	1942	4442	unpaired	internal_or_bulge	0.649023	0.526107	0.40225	0.690842	0.503992	0.302984	0.568077	0.768601	15	False	0.512521	7	True	7	True	22	False	21	False	18	False	21	False	17	False	15	13	18	0.14818
fragment01_A1036	fragment01	AGACA	1036	1036	paired	stem	0.637614	0.0489272	0.00107368	0.532179	0.256278	0.0379573	0.342857	0.420632	66	False	0.252338	57	False	53	False	54	False	65	False	67	False	68	False	65	False	65	64	66	0
fragment02	fragment0	AAACU	2189	4689	unpaired	internal_	0.626224	0.486255	0.315821	0.619661	0.365455	0.226143	0.513126	0.6837	24	False	0.439926	16	False	13	False	44	False	27	False	24	False	24	False	25	False	24	23	27	0

site_id	fragment_id	motif_seq	a_pos_fragment	a_pos_full	paired_status	local_context	exact_u1	exact_u5	exact_u10	mean_u1_pm10	mean_u5_pm10	mean_u10_pm10	final_composite_raw	final_composite_normalized	final_rank	final_top_decile	equal_weight_score	equal_weight_rank	equal_weight_top_decile	LOO_without_exact_u1_rank	LOO_without_exact_u1_top_decile	LOO_without_exact_u5_rank	LOO_without_exact_u5_top_decile	LOO_without_exact_u10_rank	LOO_without_exact_u10_top_decile	LOO_without_mean_u1_pm10_rank	LOO_without_mean_u1_pm10_top_decile	LOO_without_mean_u5_pm10_rank	LOO_without_mean_u5_pm10_top_decile	LOO_without_mean_u10_pm10_rank	LOO_without_mean_u10_pm10_top_decile	MC_median_rank	MC_Q1_rank	MC_Q3_rank	MC_top_decile_probability
_A4689	2					or_bulge																													
fragment01_A2872	fragment01	AAACA	2872	2872	unpaired	internal_or_bulge	0.624134	0.0216431	0.00409255	0.337748	0.0406201	0.00600787	0.279292	0.322422	82	False	0.172374	89	False	111	False	79	False	82	False	75	False	80	False	82	False	82	80	82	0
fragment01_A194	fragment01	AGACC	194	194	paired	stem	0.618576	0.042064	0.0113262	0.496822	0.146003	0.0342342	0.318986	0.383751	70	False	0.224838	68	False	72	False	63	False	70	False	73	False	70	False	70	False	70	69	70	0
fragment02_A2872	fragment02	AAACA	372	2872	paired	stem	0.61315	0.03485	0.0041829	0.350019	0.0421081	0.00577855	0.280736	0.324654	80	False	0.175015	87	False	106	False	81	False	80	False	76	False	79	False	80	False	80	79	81	0
fragment01_A910	fragment01	GGACA	910	910	unpaired	internal_or_bulge	0.598372	0.00864286	0.000406208	0.331574	0.0205574	0.000863292	0.263466	0.297972	84	False	0.160069	94	False	119	False	82	False	83	False	82	False	83	False	83	False	84	83	86	0
fragment02_A4562	fragment02	GGACU	2062	4562	unpaired	internal_or_bulge	0.598198	0.073795	0.0509971	0.537073	0.253114	0.127025	0.345141	0.424161	64	False	0.273367	45	False	46	False	59	False	66	False	66	False	65	False	66	False	64	63	65	0
fragment02_A4863	fragment02	UGACA	2363	4863	unpaired	internal_or_bulge	0.594477	0.196981	0.0589643	0.525963	0.124675	0.0307174	0.356106	0.441102	61	False	0.255296	55	False	42	False	65	False	63	False	62	False	62	False	61	False	61	60	62	0
fragme	fragm	UGACU	1951	1951	unpair	inter	0.587	0.020	0.004	0.451	0.088	0.007	0.2882	0.336273	77	False	0.1935	78	False	86	False	75	False	77	False	80	False	78	False	77	False	77	77	78	0

site_id	fragment_id	motif_seq	a_pos_fragment	a_pos_full	paired_status	local_context	exact_u1	exact_u5	exact_u10	mean_u1_pm10	mean_u5_pm10	mean_u10_pm10	final_composite_raw	final_composite_normalized	final_rank	final_top_decile	equal_weight_score	equal_weight_rank	equal_weight_top_decile	LOO_without_exact_u1_rank	LOO_without_exact_u1_top_decile	LOO_without_exact_u5_rank	LOO_without_exact_u5_top_decile	LOO_without_exact_u10_rank	LOO_without_exact_u10_top_decile	LOO_without_mean_u1_pm10_rank	LOO_without_mean_u1_pm10_top_decile	LOO_without_mean_u5_pm10_rank	LOO_without_mean_u5_pm10_top_decile	LOO_without_mean_u10_pm10_rank	LOO_without_mean_u10_pm10_top_decile	MC_median_rank	MC_Q1_rank	MC_Q3_rank	MC_top_decile_probability
nt01_A1951	ent01				ed	nal_or_bulge	263	8613	17995	973	896	90501	56				13																		
fragment02_A3380	fragment02	AGACA	880	3380	unpaired	internal_or_bulge	0.585236	0.0475097	0.02196	0.425034	0.0410568	0.00644716	0.287089	0.33447	78	False	0.187874	83	False	87	False	78	False	78	False	77	False	75	False	78	False	78	77	79	0
fragment02_A5144	fragment02	AAACU	2644	5144	unpaired	internal_or_bulge	0.578047	0.19521	0.0123327	0.550252	0.158502	0.0363857	0.352559	0.435622	63	False	0.255121	56	False	39	False	66	False	62	False	65	False	63	False	63	False	63	61	63	0
fragment01_A1124	fragment01	GGACC	1124	1124	paired	stem	0.572264	0.00666431	0.00178861	0.46665	0.0770243	0.00779512	0.280227	0.323868	81	False	0.188698	82	False	91	False	77	False	81	False	83	False	82	False	81	False	81	80	82	0
fragment01_A326	fragment01	AGACC	326	326	unpaired	internal_or_bulge	0.569323	0.0999661	0.0614436	0.449282	0.0749801	0.0105511	0.305749	0.3633	73	False	0.210879	73	False	65	False	76	False	73	False	74	False	73	False	73	False	73	72	73	0
fragment03_A5656	fragment03	AAACU	656	5656	paired	stem	0.567399	0.423864	0.280703	0.723598	0.505513	0.352104	0.509284	0.677764	26	False	0.475466	11	True	12	True	37	False	28	False	28	False	30	False	26	False	25	23	28	0.00053
fragment02_A4394	fragment02	AAACA	1894	4394	unpaired	hairpin_loop	0.562951	0.086207	0.078047	0.393337	0.181292	0.0404074	0.305544	0.362983	74	False	0.223712	69	False	62	False	74	False	74	False	71	False	74	False	74	False	73	72	74	0
fra	fr	AA	14	51	un	in	0.	0.	0.	0.	0.	0.	0.3	0.42	65	Fa	0.2	58	Fal	41	False	69	False	64	False	68	False	64	False	64	False	65	63	66	0

site_id	fragment_id	motif_seq	a_pos_fragment	a_pos_full	paired_status	local_context	exact_u1	exact_u5	exact_u10	mean_u1_pm10	mean_u5_pm10	mean_u10_pm10	final_composite_raw	final_composite_normalized	final_rank	final_top_decile	equal_weight_score	equal_weight_rank	equal_weight_top_decile	LOO_without_exact_u1_rank	LOO_without_exact_u1_top_decile	LOO_without_exact_u5_rank	LOO_without_exact_u5_top_decile	LOO_without_exact_u10_rank	LOO_without_exact_u10_top_decile	LOO_without_mean_u1_pm10_rank	LOO_without_mean_u1_pm10_top_decile	LOO_without_mean_u5_pm10_rank	LOO_without_mean_u5_pm10_top_decile	LOO_without_mean_u10_pm10_rank	LOO_without_mean_u10_pm10_top_decile	MC_median_rank	MC_Q1_rank	MC_Q3_rank	MC_top_decile_probability
gment03_A5144	agment03	ACU	4	44	paired	ternal_or_bulge	560481	186902	0127739	547752	160577	0389593	44305	2869		lse	51202		se																
fragment01_A803	fragment01	GAACA	803	803	unpaired	internal_or_bulge	0.555946	0.0437169	0.00192727	0.449299	0.106892	0.00784753	0.284179	0.329974	79	False	0.194271	76	False	81	False	80	False	79	False	81	False	81	False	79	False	79	79	81	0
fragment02_A5175	fragment02	GGACA	2675	5175	unpaired	internal_or_bulge	0.546967	0.0491091	0.00348227	0.496262	0.168544	0.0246244	0.296589	0.349147	75	False	0.214831	71	False	68	False	72	False	75	False	78	False	76	False	75	False	75	74	75	0
fragment03_A5175	fragment03	GGACA	175	5175	paired	stem	0.546967	0.0491091	0.00348227	0.496262	0.168544	0.0246244	0.296589	0.349147	76	False	0.214831	72	False	69	False	73	False	76	False	79	False	77	False	76	False	76	75	76	0
fragment02_A4665	fragment02	UAACA	2165	4665	unpaired	internal_or_bulge	0.540074	0.148572	0.0390478	0.509448	0.164406	0.0733177	0.326831	0.395871	67	False	0.245922	59	False	45	False	70	False	67	False	69	False	69	False	67	False	68	67	69	0
fragment02_A4637	fragment02	AAACU	2137	4637	unpaired	internal_or_bulge	0.522101	0.343724	0.00955654	0.527988	0.200241	0.0545744	0.371573	0.464999	58	False	0.276364	44	False	30	False	71	False	57	False	60	False	59	False	58	False	58	53	60	0
fragment01_A1	fragment01	GGACA	1063	1063	paired	stem	0.517512	0.0119761	0.00043011	0.359293	0.0384019	0.00157646	0.241979	0.264774	88	False	0.154865	96	False	110	False	86	False	88	False	86	False	86	False	88	False	88	87	89	0

site_id	fragment_id	motif_seq	a_pos_fragment	a_pos_full	paired_status	local_context	exact_u1	exact_u5	exact_u10	mean_u1_pm10	mean_u5_pm10	mean_u10_pm10	final_composite_raw	final_composite_normalized	final_rank	final_top_decile	equal_weight_score	equal_weight_rank	equal_weight_top_decile	LOO_without_exact_u1_rank	LOO_without_exact_u1_top_decile	LOO_without_exact_u5_rank	LOO_without_exact_u5_top_decile	LOO_without_exact_u10_rank	LOO_without_exact_u10_top_decile	LOO_without_mean_u1_pm10_rank	LOO_without_mean_u1_pm10_top_decile	LOO_without_mean_u5_pm10_rank	LOO_without_mean_u5_pm10_top_decile	LOO_without_mean_u10_pm10_rank	LOO_without_mean_u10_pm10_top_decile	MC_median_rank	MC_Q1_rank	MC_Q3_rank	MC_top_decile_probability
063																																			
fragment02_A4823	fragment02	AAACA	2323	4823	unpaired	internal_or_bulge	0.515899	0.471832	0.373044	0.673336	0.432669	0.342547	0.497225	0.659133	27	False	0.468225	12	True	11	True	48	False	33	False	32	False	31	False	29	False	27	25	32	0.00025
fragment01_A1235	fragment01	GAACC	1235	1235	unpaired	internal_or_bulge	0.498515	0.366724	0.00300288	0.634146	0.251548	0.0842918	0.390953	0.494941	53	False	0.306371	37	False	24	False	67	False	53	False	57	False	55	False	54	False	53	44	56	0
fragment01_A1965	fragment01	UAACA	1965	1965	unpaired	internal_or_bulge	0.479567	0.0821921	0.0153718	0.375418	0.0838883	0.0110357	0.255187	0.28518	85	False	0.174578	88	False	82	False	87	False	85	False	85	False	85	False	85	False	85	85	86	0
fragment01_A2470	fragment01	AGACU	2470	2470	unpaired	internal_or_bulge	0.463393	0.0617963	0.0490828	0.432897	0.139742	0.056572	0.264282	0.299233	83	False	0.20058	74	False	73	False	83	False	84	False	84	False	84	False	84	False	84	83	84	0
fragment02_A4310	fragment02	AGACC	1810	4310	unpaired	internal_or_bulge	0.431967	0.0518206	0.00485628	0.257464	0.0265099	0.00351562	0.206076	0.209303	95	False	0.129356	106	False	117	False	92	False	95	False	88	False	94	False	95	False	95	92	95	0
fragment01_A1899	fragment01	AAACA	1899	1899	unpaired	internal_or_bulge	0.427478	0.310253	0.00955506	0.467165	0.164799	0.04246	0.316814	0.380395	71	False	0.236952	63	False	36	False	85	False	71	False	70	False	72	False	71	False	70	68	74	0
fragme	fragm	GGACA	1300	1300	unpair	hairpi	0.417	0.016	0.001	0.575	0.145	0.007	0.2515	0.279591	86	False	0.1938	77	False	67	False	84	False	86	False	90	False	87	False	86	False	86	85	87	0

site_id	fragment_id	motif_seq	a_pos_fragment	a_pos_full	paired_status	local_context	exact_u1	exact_u5	exact_u10	mean_u1_pm10	mean_u5_pm10	mean_u10_pm10	final_composite_raw	final_composite_normalized	final_rank	final_top_decile	equal_weight_score	equal_weight_rank	equal_weight_top_decile	LOO_without_exact_u1_rank	LOO_without_exact_u1_top_decile	LOO_without_exact_u5_rank	LOO_without_exact_u5_top_decile	LOO_without_exact_u10_rank	LOO_without_exact_u10_top_decile	LOO_without_mean_u1_pm10_rank	LOO_without_mean_u1_pm10_top_decile	LOO_without_mean_u5_pm10_rank	LOO_without_mean_u5_pm10_top_decile	LOO_without_mean_u10_pm10_rank	LOO_without_mean_u10_pm10_top_decile	MC_median_rank	MC_Q1_rank	MC_Q3_rank	MC_top_decile_probability
nt01_A1300	ent01				ed	n_loop	539	6397	15574	074	11	65517	69				62																		
fragment01_A1444	fragment01	AAACC	1444	1444	unpaired	internal_or_bulge	0.406937	0.347631	0.211316	0.611526	0.336805	0.183645	0.385059	0.485835	55	False	0.349643	28	False	23	False	68	False	58	False	59	False	60	False	57	False	55	45	58	0
fragment02_A2957	fragment02	GAACU	457	2957	unpaired	internal_or_bulge	0.383192	0.0629794	0.00249285	0.352042	0.0626001	0.0212961	0.210242	0.215574	94	False	0.147434	98	False	98	False	91	False	94	False	92	False	92	False	94	False	93	92	94	0
fragment02_A3512	fragment02	AGACA	1012	3512	unpaired	internal_or_bulge	0.353452	0.114023	0.00542288	0.431544	0.0897979	0.00959504	0.226948	0.24155	90	False	0.167306	92	False	71	False	90	False	90	False	91	False	90	False	89	False	90	89	90	0
fragment03_A5039	fragment03	GGACA	39	5039	paired	stem	0.350389	0.00444414	1.37995e-05	0.518705	0.125998	0.0192816	0.215118	0.223274	91	False	0.169805	91	False	79	False	88	False	91	False	95	False	95	False	91	False	92	91	94	0
fragment02_A5089	fragment02	UGACU	2589	5089	paired	stem	0.336414	0.0622111	0.00939425	0.454345	0.110421	0.0267374	0.214768	0.222732	92	False	0.166587	93	False	77	False	89	False	92	False	93	False	93	False	92	False	92	92	93	0
fragment02_A3868	fragment02	GGACA	1368	3868	paired	stem	0.322804	0.0332932	0.00612408	0.330336	0.0822869	0.00847689	0.18012	0.169201	99	False	0.130554	105	False	105	False	99	False	99	False	96	False	99	False	99	False	99	97	102	0
fragm	frag	GGAC	1370	1370	unpa	inte	0.31	0.11	0.00	0.43	0.05	0.00	0.211	0.217866	93	Fals	0.155	95	False	74	False	94	False	93	False	94	False	91	False	93	False	93	92	95	0

site_id	fragment_id	motif_seq	a_pos_fragment	a_pos_full	paired_status	local_context	exact_u1	exact_u5	exact_u10	mean_u1_pm10	mean_u5_pm10	mean_u10_pm10	final_composite_raw	final_composite_normalized	final_rank	final_top_decile	equal_weight_score	equal_weight_rank	equal_weight_top_decile	LOO_without_exact_u1_rank	LOO_without_exact_u1_top_decile	LOO_without_exact_u5_rank	LOO_without_exact_u5_top_decile	LOO_without_exact_u10_rank	LOO_without_exact_u10_top_decile	LOO_without_mean_u1_pm10_rank	LOO_without_mean_u1_pm10_top_decile	LOO_without_mean_u5_pm10_rank	LOO_without_mean_u5_pm10_top_decile	LOO_without_mean_u10_pm10_rank	LOO_without_mean_u10_pm10_top_decile	MC_median_rank	MC_Q1_rank	MC_Q3_rank	MC_top_decile_probability
ent01_A1370	ment01	A			ired	rnal_or_bulge	6088	7143	0468737	9041	70743	182534	618			e	273																		
fragment01_A2036	fragment01	UGACA	2036	2036	unpaired	internal_or_bulge	0.309246	0.023695	8.0048e-05	0.381178	0.0590268	0.00127824	0.177311	0.164861	101	False	0.129084	107	False	102	False	100	False	101	False	98	False	100	False	101	False	101	100	103	0
fragment02_A3637	fragment02	GGACA	1137	3637	paired	stem	0.307866	0.00107703	1.6110e-05	0.273989	0.0107918	0.000385069	0.150221	0.123006	111	False	0.0990208	116	False	120	False	105	False	111	False	102	False	106	False	110	False	111	107	112	0
fragment02_A4741	fragment02	UGACA	2241	4741	unpaired	internal_or_bulge	0.304877	0.0129586	0.000169341	0.444281	0.0720116	0.00586344	0.1841	0.17535	97	False	0.140027	100	False	94	False	95	False	97	False	100	False	98	False	97	False	98	97	99	0
fragment01_A1688	fragment01	GAACU	1688	1688	paired	stem	0.295626	0.0390283	0.000461909	0.434002	0.0465071	0.00688932	0.183368	0.174219	98	False	0.137086	103	False	92	False	98	False	98	False	99	False	97	False	98	False	98	97	99	0
fragment01_A2290	fragment01	AGACU	2290	2290	paired	stem	0.283735	0.243181	0.00174747	0.492606	0.143242	0.00601407	0.248636	0.275059	87	False	0.194826	75	False	40	False	93	False	87	False	87	False	88	False	87	False	87	84	88	0
fragment02_A4019	fragment02	GGACA	1519	4019	paired	stem	0.282814	0.00140164	7.4981e-05	0.400698	0.0727599	0.00439613	0.166943	0.148843	105	False	0.127024	109	False	104	False	101	False	103	False	104	False	103	False	103	False	104	102	106	0
frag	fra	AGA	227	477	unp	int	0.2	0.2	0.0	0.4	0.1	0.0	0.22	0.2452	89	Fal	0.18	84	False	49	False	97	False	89	False	89	False	89	False	90	False	89	88	90	0

site_id	fragment_id	motif_seq	a_pos_fragment	a_pos_full	paired_status	local_context	exact_u1	exact_u5	exact_u10	mean_u1_pm10	mean_u5_pm10	mean_u10_pm10	final_composite_raw	final_composite_normalized	final_rank	final_top_decile	equal_weight_score	equal_weight_rank	equal_weight_top_decile	LOO_without_exact_u1_rank	LOO_without_exact_u1_top_decile	LOO_without_exact_u5_rank	LOO_without_exact_u5_top_decile	LOO_without_exact_u10_rank	LOO_without_exact_u10_top_decile	LOO_without_mean_u1_pm10_rank	LOO_without_mean_u1_pm10_top_decile	LOO_without_mean_u5_pm10_rank	LOO_without_mean_u5_pm10_top_decile	LOO_without_mean_u10_pm10_rank	LOO_without_mean_u10_pm10_top_decile	MC_median_rank	MC_Q1_rank	MC_Q3_rank	MC_top_decile_probability
ment02_A4778	gment02	CU	8	8	aired	ernal_or_bulge	74006	12652	278248	23513	08571	622574	9345	54		se	4804																		
fragment02_A5039	fragment02	GGACA	2539	5039	paired	stem	0.2676889	0.001272889	1.53553e-05	0.4906994	0.1102266	0.008118864	0.1790048	0.167544	100	False	0.1463443	99	False	85	False	96	False	100	False	107	False	101	False	100	False	100	99	101	0
fragment02_A3532	fragment02	GGACA	1032	3532	paired	stem	0.2502884	0.0003132442	3.8884e-07	0.348899	0.09692273	0.000892237	0.1497764	0.1223	112	False	0.1162234	114	False	108	False	106	False	112	False	108	False	110	False	111	False	111	110	111	0
fragment03_A5089	fragment03	UGACU	89	5089	paired	stem	0.2473556	0.04717702	0.008540009	0.4104434	0.06741677	0.01051179	0.1680054	0.150559	104	False	0.1319906	104	False	89	False	104	False	102	False	105	False	102	False	102	False	104	103	105	0
fragment01_A1408	fragment01	AAACC	1408	1408	paired	stem	0.2383357	0.1677704	0.0009610089	0.3959983	0.03319905	0.003639976	0.1883446	0.18191	96	False	0.1399733	101	False	70	False	107	False	96	False	97	False	96	False	96	False	96	96	99	0
fragment01_A1141	fragment01	AAACA	1141	1141	paired	stem	0.2324441	0.04824777	0.02362245	0.3177667	0.12108	0.02564668	0.1568334	0.133224	107	False	0.1281335	108	False	99	False	108	False	108	False	101	False	107	False	107	False	108	107	109	0
fragment01_A1145	fragment01	AGACC	1145	1145	paired	stem	0.2283533	0.08154451	0.006742254	0.3165884	0.0671784	0.01936997	0.1561559	0.13218	108	False	0.1199663	113	False	97	False	114	False	107	False	103	False	105	False	108	False	108	107	110	0
fragmen	fragme	GAACU	2957	2957	unpaire	intern	0.1982	0.0985	0.0060	0.3915	0.0961	0.0335	0.16465	0.145305	106	False	0.13734	102	False	78	False	111	False	106	False	106	False	104	False	106	False	105	104	106	0

site_id	fragment_id	motif_seq	a_pos_fragment	a_pos_full	paired_status	local_context	exact_u1	exact_u5	exact_u10	mean_u1_pm10	mean_u5_pm10	mean_u10_pm10	final_composite_raw	final_composite_normalized	final_rank	final_top_decile	equal_weight_score	equal_weight_rank	equal_weight_top_decile	LOO_without_exact_u1_rank	LOO_without_exact_u1_top_decile	LOO_without_exact_u5_rank	LOO_without_exact_u5_top_decile	LOO_without_exact_u10_rank	LOO_without_exact_u10_top_decile	LOO_without_mean_u1_pm10_rank	LOO_without_mean_u1_pm10_top_decile	LOO_without_mean_u5_pm10_rank	LOO_without_mean_u5_pm10_top_decile	LOO_without_mean_u10_pm10_rank	LOO_without_mean_u10_pm10_top_decile	MC_median_rank	MC_Q1_rank	MC_Q3_rank	MC_top_decile_probability
t01_A2957	nt01				d	al_or_bulge	57	574	1218	51	067	984	4				7																		
fragment01_A2671	fragment01	AGACA	2671	2671	paired	stem	0.178846	0.00318291	0.00175739	0.459346	0.0627575	0.0148825	0.139489	0.106426	113	False	0.120129	111	False	95	False	112	False	113	False	113	False	112	False	113	False	113	112	113	0
fragment02_A2671	fragment02	AGACA	171	2671	paired	stem	0.178846	0.00318291	0.00175739	0.459346	0.0627575	0.0148825	0.139489	0.106426	114	False	0.120129	112	False	96	False	113	False	114	False	114	False	113	False	114	False	114	113	114	0
fragment02_A4290	fragment02	GGACC	1790	4290	paired	stem	0.16159	0.00159606	7.8082e-05	0.437555	0.0201194	0.000444992	0.124631	0.0834691	115	False	0.103564	115	False	103	False	115	False	115	False	115	False	115	False	115	False	115	115	115	0
fragment01_A1704	fragment01	AGACA	1704	1704	paired	stem	0.134297	0.0586769	0.035186	0.472674	0.185998	0.0168682	0.155536	0.131218	109	False	0.150617	97	False	61	False	109	False	109	False	112	False	111	False	109	False	109	107	111	0
fragment02_A3090	fragment02	AGACU	590	3090	paired	stem	0.12366	0.0199677	0.00482758	0.341637	0.0346492	0.0073174	0.103853	0.0513668	117	False	0.0887456	117	False	112	False	117	False	117	False	116	False	116	False	117	False	117	116	117	0
fragment02_A3900	fragment02	GAACC	1400	3900	paired	stem	0.123637	0.0427823	0.00516891	0.401073	0.275441	0.183749	0.151378	0.124794	110	False	0.171975	90	False	63	False	110	False	110	False	109	False	114	False	112	False	110	108	112	0
fragment02	fragment0	UGACA	2696	5196	paired	stem	0.118079	0.0437139	0.0445687	0.52868	0.25717	0.0922472	0.169973	0.153523	102	False	0.184774	85	False	50	False	102	False	104	False	110	False	108	False	104	False	102	99	106	0

site_id	fragment_id	motif_seq	a_pos_fragment	a_pos_full	paired_status	local_context	exact_u1	exact_u5	exact_u10	mean_u1_pm10	mean_u5_pm10	mean_u10_pm10	final_composite_raw	final_composite_normalized	final_rank	final_top_decile	equal_weight_score	equal_weight_rank	equal_weight_top_decile	LOO_without_exact_u1_rank	LOO_without_exact_u1_top_decile	LOO_without_exact_u5_rank	LOO_without_exact_u5_top_decile	LOO_without_exact_u10_rank	LOO_without_exact_u10_top_decile	LOO_without_mean_u1_pm10_rank	LOO_without_mean_u1_pm10_top_decile	LOO_without_mean_u5_pm10_rank	LOO_without_mean_u5_pm10_top_decile	LOO_without_mean_u10_pm10_rank	LOO_without_mean_u10_pm10_top_decile	MC_median_rank	MC_Q1_rank	MC_Q3_rank	MC_top_decile_probability
_A5196	2																																		
fragment03_A5196	fragment03	UGACA	196	5196	paired	stem	0.118079	0.0437139	0.0445687	0.552868	0.25717	0.0922472	0.169973	0.153523	103	False	0.184774	86	False	51	False	103	False	105	False	111	False	109	False	105	False	103	100	107	0
fragment01_A2845	fragment01	UGACA	2845	2845	paired	stem	0.0442297	0.00341225	2.0635e-05	0.324495	0.0950743	0.00101125	0.0745677	0.00612101	118	False	0.0780405	118	False	113	False	118	False	118	False	118	False	119	False	118	False	118	118	118	0
fragment02_A2845	fragment02	UGACA	345	2845	paired	stem	0.0442297	0.00341225	2.0635e-05	0.322977	0.0950535	0.00101125	0.0743338	0.005766	119	False	0.0777784	119	False	114	False	119	False	119	False	119	False	120	False	119	False	119	119	119	0
fragment02_A3615	fragment02	UGACC	115	3615	paired	stem	0.0271228	0.00629496	0.00614905	0.373883	0.025994	0.00484961	0.0706006	0	120	False	0.0740489	120	False	109	False	120	False	120	False	120	False	118	False	120	False	120	120	120	0
fragment01_A2113	fragment01	UGACA	2113	2113	unpaired	internal_or_bulge	0.0257671	0.00227906	0.00166715	0.531072	0.17193	0.00778071	0.106998	0.056226	116	False	0.123416	110	False	76	False	116	False	116	False	117	False	117	False	116	False	116	116	117	0

Notes. Sites represented in overlapping fragments are intentionally retained as separate fragment-level observations; therefore, the same full-transcript coordinate may occur more than once. The final composite score used weights 0.35, 0.25, 0.10, 0.15, 0.10, and 0.05 for exact_u1, exact_u5, exact_u10, mean_u1_pm10, mean_u5_pm10, and mean_u10_pm10, respectively. The original top-decile subset comprised the 12 highest-ranked observations (10% of n = 120).

Supplementary Table S2. Structural accessibility-ranking robustness under equal-weight, leave-one-component-out, and Monte Carlo sensitivity analyses.

analysis	excluded_component	spearman_rho_vs_final	top_decile_retained_n	top_decile_total
Equal weighting		0.974032	7	12
Leave-one-out: exact_u1	exact_u1	0.78582	7	12
Leave-one-out: exact_u5	exact_u5	0.97224	8	12
Leave-one-out: exact_u10	exact_u10	0.998819	12	12
Leave-one-out: mean_u1_pm10	mean_u1_pm10	0.995854	12	12
Leave-one-out: mean_u5_pm10	mean_u5_pm10	0.997514	12	12
Leave-one-out: mean_u10_pm10	mean_u10_pm10	0.999674	12	12

Monte Carlo summary (100,000 perturbations).

Metric	Result
Dirichlet concentration	50
Median Spearman ρ	0.997201
Spearman ρ, IQR	0.994687–0.998625
Median original top-decile observations retained	11/12
Top-decile retention, IQR	11/12–12/12

Notes. Spearman ρ compares each alternative ranking with the final structural ranking. top_decile_retained_n is the number of observations from the original 12-observation top-decile set retained under the alternative analysis. Monte Carlo weights were sampled from a Dirichlet distribution centered on the final weight vector (0.35, 0.25, 0.10, 0.15, 0.10, 0.05) with concentration parameter 50.
